# Effect of oral faecal microbiota transplantation intervention for children with autism spectrum disorder: A randomised, double‐blind, placebo‐controlled trial

**DOI:** 10.1002/ctm2.70006

**Published:** 2024-08-26

**Authors:** Lin Wan, Huan Wang, Yan Liang, Xun Zhang, Xinyun Yao, Gang Zhu, Jun Cai, Guoyin Liu, Xinting Liu, Qianqian Niu, Siwen Li, Bo Zhang, Jing Gao, Jing Wang, Xiuyu Shi, Linyan Hu, Xiaoyan Liu, Zhiyong Zou, Guang Yang

**Affiliations:** ^1^ Senior Department of Pediatrics The Seventh Medical Centre of PLA General Hospital Beijing China; ^2^ Department of Pediatrics The First Medical Centre Chinese PLA General Hospital Beijing China; ^3^ National Health Commission Key Laboratory of Reproductive Health Institute of Child and Adolescent Health School of Public Health Peking University Beijing China; ^4^ Department of Pharmacy PLA General Hospital Beijing China; ^5^ Hypertension Centre, Fuwai Hospital State Key Laboratory of Cardiovascular Disease National Center for Cardiovascular Diseases Peking Union Medical College & Chinese Academy of Medical Sciences Beijing China; ^6^ Department of Neurology and ICCTR Biostatistics and Research Design Centre Boston Children's Hospital Harvard Medical School Boston Massachusetts USA; ^7^ Heart Centre and Beijing Key Laboratory of Hypertension Beijing Chao‐Yang Hospital Capital Medical University Beijing China; ^8^ The Second School of Clinical Medicine Southern Medical University Guangzhou China

To the Editor:

We conducted a randomised, double‐blind, placebo‐controlled trial of an oral faecal microbiota transplantation (FMT) intervention in children with autism spectrum disorder (ASD), and observed that there were differences in pre‐ and post‐FMT intervention changes in the social domain scores of the Vineland Adaptive Behaviour Scale, third version (Vineland‐3), with no severe adverse events (AEs) related to FMT occurring.

Multiple studies have documented variations in the gut microbiota (GM) between children with ASD and typically developing children.^1^, ^2^ Furthermore, specific gut microbes may modulate ASD‐related behaviour through their metabolites.^1^ However, another study proposed that the GM is not the central driver of ASD symptoms; rather, the presence of restricted interests associated with ASD and a more limited dietary variety have been linked to reduced diversity in the GM.^3^ The relationship between the GM and ASD remains enigmatic and complex as the age‐old philosophical conundrum that has long been debated: which came first, the chicken or the egg? Some open‐label studies^4^, ^5^, ^6^ have demonstrated that FMT improved the core symptoms of patients with ASD, though these studies lacked comparable control groups, which may lead to disregard of potential placebo effects on efficacy evaluation. Therefore, we conducted this study.

A total of 103 eligible participants (Figure 1) were enrolled and randomised to receive either FMT or a placebo. These agents were administered during two 6‐day periods in the hospital, the first occurring in the initial week and the second in the fifth week of the study. Patients were not hospitalised during the interval between treatments. FMT capsules from five healthy donors were randomly provided to 8, 8, 11, 7 and 18 patients, respectively, with each patient receiving capsules from the same donor throughout the treatment process. The Social Responsiveness Scale, Second Edition (SRS‐2), Vineland‐3, and Autism Behaviour Checklist (ABC) scales were administered to measure outcomes before the start of the treatment as well as at Weeks 9 and 17 of the experiment for repeated measures (see Appendix S1).

The primary outcome was the difference in the change in the SRS‐2 *T*‐score between groups from baseline to Week 9 or Week 17, analysed using a mixed model for repeated measures. The secondary outcomes included Vineland‐3 and ABC scores. Sensitivity analysis was conducted by comparing the changes in the scores of these scales between the FMT group with a donor gut microbiota colonisation rate of ≥20% or less than 20% and the placebo group. AEs were assessed (see Appendix S1).

No notable between‐group differences were detected between the FMT (*n* = 52) and placebo (*n* = 51) groups in terms of demographic and baseline characteristics or scales (Appendix S1; Tables S1 and S2).

For the primary outcome, there were no significant differences in the SRS‐2 *T*‐scores between the two groups at baseline, Week 9 or Week 17 (Appendix S1; Figure S2). In the FMT group, the SRS‐2 *T*‐score decreased significantly from 78.33 at baseline to 74.59 at Week 17 (*p* = .03; Appendix S1; Table S3). From baseline to Week 17, significant reductions were observed in the *T*‐scores of SRS‐2 and its domains in both groups; however, the between‐group differences in the changes in *T*‐scores were not statistically significant (Table 1).

**TABLE 1:** Changes in scores in the FMT and placebo groups from baseline to Week 9 and Week 17.

	Baseline to Week 9					Baseline to Week 17				
Scores	FMT (*n* = 52)	Placebo (*n* = 51)	Difference (95% CI)	*p*‐Value	Cohen's *d* (95% CI)	FMT (*n* = 52)	Placebo (*n* = 51)	Difference (95% CI)	*p*‐Value	Cohen's *d* (95% CI)
**Primary outcome**										
**SRS‐2 *T*‐score**	−1.43 (1.17)	−1.80 (1.18)	.37 (−2.88, 3.63)	.821	.04 (−.34, .43)	−3.74 (1.15)	−2.26 (1.16)	−1.49 (−4.69, 1.71)	.362	−.18 (−.57, .21)
Consciousness domain	−2.06 (1.68)	−1.89 (1.70)	−.17 (−4.85, 4.52)	.944	−.01 (−.40, .37)	−2.51 (1.83)	−1.11 (1.85)	−1.39 (−6.50, 3.72)	.593	−.11 (−.49, .28)
Cognition domain	−1.19 (1.18)	−.11 (1.19)	−1.08 (−4.36, 2.19)	.517	−.13 (−.51, .26)	−1.60 (1.22)	−.10 (1.23)	−1.50 (−4.9, 1.91)	.389	−.17 (−.56, .21)
Communication domain	.22 (1.39)	−2.10 (1.40)	2.32 (−1.55, 6.19)	.241	.23 (−.16, .62)	−2.92 (1.30)	−1.74 (1.31)	−.18 (−4.80, 2.44)	.524	−.13 (−.51, .26)
Motivation domain	−.86 (1.23)	−.87 (1.25)	.01 (−3.43, 3.45)	.994	.00 (−.39, .39)	−3.95 (1.71)	−1.71 (1.72)	−2.23 (−6.99, 2.52)	.358	−.18 (−.57, .20)
Behavioural styles domain	−3.76 (1.56)	−2.20 (1.58)	−1.56 (−5.91, 2.80)	.483	−.14 (−.53, .25)	−5.63 (1.57)	−4.63 (1.59)	−1.00 (−5.38, 3.37)	.654	−.09 (−.48, .30)
**Secondary outcomes**										
**Vineland‐3 composite score**	1.51 (.93)	1.30 (.94)	.21 (−2.38, 2.80)	.873	.03 (−.35, .42)	4.45 (1.08)	2.37 (1.09)	2.08 (−.94, 5.10)	.177	.27 (−.12, .66)
Communication domain	2.24 (1.22)	2.28 (1.24)	−.04 (−3.45, 3.36)	.980	−.00 (−.39, .38)	3.10 (1.39)	4.20 (1.40)	−1.09 (−4.96, 2.77)	.579	−.11 (−.50, .28)
Daily living skills domain	−.29 (1.54)	1.87 (1.56)	−2.17 (−6.46, 2.13)	.323	−.20 (−.58, .19)	2.66 (1.36)	1.53 (1.38)	1.13 (−2.67, 4.93)	.561	.12 (−.27, .50)
Socialisation domain	3.94 (1.73)	1.27 (1.75)	2.67 (−2.16, 7.50)	.279	.22 (−.17, .60)	**8.77 (1.70)**	**3.73 (1.72)**	**5.05 (.31, 9.79)**	**.037**	**.41 (.02, .81)**
**ABC score**	−12.62 (3.01)	−11.24 (3.04)	−1.38 (−9.76, 7.00)	.747	−.06 (−.45, .32)	−15.48 (3.28)	−18.14 (3.31)	2.66 (−6.48, 11.79)	.569	.11 (−.27, .50)
Sensory domain	−2.71 (.92)	−.33 (.93)	−2.38 (−4.94, .19)	.069	−.36 (−.75, .03)	−3.06 (.85)	−2.69 (.86)	−.37 (−2.75, 2.01)	.760	−.06 (−.45, .33)
Relating domain	−3.42 (1.08)	−3.00 (1.09)	−.42 (−3.43, 2.59)	.783	−.05 (−.44, .33)	−3.21 (1.23)	−4.29 (1.25)	1.08 (−2.36, 4.52)	.537	.12 (−.26, .51)
Body domain	−2.29 (.88)	−.61 (.89)	−1.68 (−4.12, .76)	.177	−.27 (−.66, .12)	−2.44 (.80)	−2.75 (.81)	.30 (−1.93, 2.53)	.790	.05 (−.33, .44)
Language domain	−3.63 (.97)	−3.33 (.98)	−.30 (−3.01, 2.41)	.827	−.04 (−.43, .34)	−3.69 (1.17)	−4.69 (1.18)	.99 (−2.26, 4.25)	.550	.12 (−.27, .51)
Social and self‐help domain	−2.27 (.75)	−1.84 (.75)	−.43 (−2.51, 1.66)	.688	−.08 (−.47, .31)	−3.42 (.84)	−3.37 (.85)	−.05 (−2.39, 2.29)	.966	−.01 (−.39, .38)

*Note*: Data are presented as least‐square means with standard errors after adjustment for sex, age and baseline Autism Diagnostic Observation Schedule score.

Abbreviations: ABC, Autism Behaviour Checklist; CI, confidence interval; FMT, faecal microbiota transplantation; SRS, Social Responsiveness Scale.

Regarding the secondary outcome, there were no significant differences in the ABC scores between the two groups at baseline, Week 9 or Week 17 (Appendix S1; Figure S4). The scores for both the FMT and placebo groups showed a reduction from baseline to Weeks 9 and 17 (Appendix S1; Table S3); however, there were no significant between‐group differences (Table 1). Regarding Vineland‐3 scores as a secondary outcome, there were no significant differences in the scores between the two groups at baseline, Week 9 or Week 17 (Appendix S1; Figure S3). After 17 weeks of treatment, the mean improvement in Vineland‐3 symptoms was reported in both the FMT and placebo groups (Appendix S1; Table S3). Notably, a statistically significant disparity was observed in the alteration of socialisation domain score from baseline to Week 17 between the FMT and the placebo groups (difference = 5.05, 95% confidence interval [CI]: .31–9.79; Table 1). Additionally, compared with the placebo group (.55), the score in the play and leisure subdomain was significantly greater in the FMT group (1.91) from baseline to Week 17 (difference = 1.36, 95% CI: .19–2.53; Appendix S1; Figure S1; Table S4).

Regarding alpha diversity, substantial differences were noted in the Shannon, Simpson and Invsimpson indices at Week 5 between the two groups (Appendix S1; Figures S5A), whereas the beta diversity (the Bray–Curtis distance from baseline to Week 9) in the FMT group was significantly greater than that in the placebo group (Appendix S1; Figure S5B).

In the sensitivity analysis, 30 out of the 52 participants in the FMT group had a colonisation rate ≥20% at Week 17. Specifically, participants with a higher colonisation rate (≥20%) presented greater improvements in the domains of the Vineland‐3 scale than the placebo group at Week 17 (Table 2). Changes in the Vineland‐3 subdomain scores in the FMT (according to the colonisation rate) and placebo groups are presented in Table S5 (Appendix S1).

**TABLE 2:** Changes in scores in the FMT and placebo groups from baseline to Week 9 and Week 17, based on the colonisation rate at Week 17.

	Baseline to Week 9					Baseline to Week 17				
Scores	FMT	Placebo (*n* = 51)	Difference (95% CI)	*p*‐Value	Cohen's d (95% CI)	FMT	Placebo (*n* = 51)	Difference (95% CI)	*p*‐Value	Cohen's *d* (95% CI)
**Colonisation rate <20% (*n* = 22 for FMT)**										
**Primary outcome**										
**SRS‐2 *T*‐score**	−3.05 (1.78)	−1.47 (1.17)	−1.57 (−5.76, 2.61)	.461	−.19 (−.69, 3.12)	−4.23 (1.71)	−2.31 (1.12)	−1.91 (−5.92, 2.09)	.349	−.24 (−.74, .26)
Consciousness domain	−3.18 (2.57)	−1.82 (1.69)	−1.36 (−7.38, 4.66)	.658	−.11 (−.61, .39)	−5.00 (2.70)	−.33 (1.77)	−4.67 (−1.99, 1.66)	.148	−.37 (−.87, .13)
Cognition domain	−5.55 (1.94)	.22 (1.28)	**−5.76 (−1.32, −1.20)**	.013	**−.63 (−.14, −.12)**	−3.73 (1.74)	−.41 (1.14)	−3.32 (−7.4, .76)	.111	−.41 (−.91, .1)
Communication domain	−1.32 (2.01)	−2.22 (1.32)	.90 (−3.82, 5.61)	.709	.10 (−.40, .60)	−4.36 (1.93)	−1.94 (1.27)	−2.42 (−6.96, 2.11)	.295	−.27 (−.77, .23)
Motivation domain	−4.23 (1.85)	−.24 (1.21)	−3.99 (−8.32, .34)	.071	−.46 (−.97, .04)	−3.36 (2.25)	−1.25 (1.48)	−2.11 (−7.39, 3.17)	.434	−.20 (−.70, .30)
Behavioural styles domain	−4.41 (2.84)	−1.76 (1.86)	−2.64 (−9.30, 4.01)	.436	−.2 (−.70, .30)	−5.86 (2.59)	−5.49 (1.70)	−.37 (−6.45, 5.70)	.904	−.03 (−.53, .47)
**Secondary outcomes**										
**Vineland‐3 composite score**	−.18 (1.37)	.92 (.90)	−1.10 (−4.31, 2.11)	.500	−.17 (−.67, .33)	.73 (1.52)	2.37 (1.00)	−1.65 (−5.2, 1.91)	.364	−.23 (−.73, .27)
Communication domain	−.27 (1.72)	1.88 (1.13)	−2.16 (−6.19, 1.88)	.295	−.27 (−.77, .24)	−1.59 (2.19)	4.20 (1.44)	**−5.79 (−1.91, −.66)**	.027	**−.56 (−1.07, −.06)**
Daily living skills domain	−2.09 (2.02)	1.43 (1.33)	−3.52 (−8.26, 1.21)	.145	−.37 (−.87, .13)	−2.14 (2.00)	1.53 (1.31)	−3.67 (−8.36, 1.02)	.126	−.39 (−.90, .11)
Socialisation domain	3.36 (2.67)	.86 (1.75)	2.50 (−3.76, 8.76)	.434	.20 (−.30, .70)	6.95 (2.77)	3.73 (1.82)	3.23 (−3.28, 9.73)	.331	.25 (−.25, .75)
**ABC score**	−13.36 (4.46)	−11.24 (2.93)	−2.13 (−12.58, 8.32)	.690	−.10 (−.60, .40)	−14.64 (5.02)	−18.14 (3.30)	3.50 (−8.28, 15.28)	.560	.15 (−.35, .65)
Sensory domain	−3.32 (1.41)	−.33 (.93)	−2.98 (−6.30, .33)	.078	−.45 (−.96, .05)	−3.68 (1.34)	−2.69 (.88)	−1.00 (−4.13, 2.14)	.534	−.16 (−.66, .34)
Relating domain	−4.73 (1.71)	−3.00 (1.12)	−1.73 (−5.74, 2.29)	.399	−.22 (−.72, .29)	−3.09 (1.97)	−4.29 (1.30)	1.20 (−3.43, 5.83)	.610	.13 (−.37, .63)
Body domain	−1.55 (1.27)	−.61 (.83)	−.94 (−3.91, 2.04)	.537	−.16 (−.66, .34)	.27 (1.09)	−2.75 (.72)	**3.02 (.45, 5.58)**	.021	**.59 (.08, 1.10)**
Language domain	−4.36 (1.52)	−3.33 (1.00)	−1.03 (−4.59, 2.53)	.571	−.14 (−.64, .36)	−5.05 (1.83)	−4.69 (1.20)	−.36 (−4.65, 3.93)	.870	−.04 (−.54, .46)
Social and self‐help domain	−1.73 (1.07)	−1.84 (.70)	.12 (−2.40, 2.63)	.928	.02 (−.48, .52)	−3.05 (1.28)	−3.37 (.84)	.33 (−2.67, 3.32)	.830	.05 (−.45, .55)
**Colonisation rate ≥20% (*n* = 30 for FMT)**										
**Primary outcome**										
**SRS‐2 *T*‐score**	−.43 (1.58)	−1.47 (1.21)	1.04 (−2.86, 4.93)	.601	.12 (−.33, .57)	−3.60 (1.56)	−2.31 (1.20)	−1.29 (−5.14, 2.56)	.512	−.15 (−.60, .30)
Consciousness domain	−1.57 (2.22)	−1.82 (1.70)	.26 (−5.22, 5.74)	.927	.02 (−.43, .47)	−2.70 (2.45)	−.33 (1.88)	−2.37 (−8.42, 3.69)	.444	−.18 (−.63, .28)
Cognition domain	.07 (1.55)	.22 (1.19)	−.15 (−3.99, 3.69)	.939	−.02 (−.47, .43)	−1.10 (1.72)	−.41 (1.32)	−.69 (−4.94, 3.56)	.751	−.07 (−.52, .38)
Communication domain	.70 (1.72)	−2.22 (1.32)	2.92 (−1.32, 7.15)	.177	.31 (−.14, .76)	−2.37 (1.78)	−1.94 (1.36)	−.43 (−4.82, 3.97)	.849	−.04 (−.50, .41)
Motivation domain	.23 (1.69)	−.24 (1.30)	.47 (−3.72, 4.66)	.826	.05 (−.40, .50)	−5.07 (2.10)	−1.25 (1.61)	−3.81 (−9.01, 1.38)	.150	−.33 (−.79, .12)
Behavioural styles domain	−2.17 (2.32)	−1.76 (1.78)	−.40 (−6.14, 5.34)	.891	−.03 (−.48, .42)	−5.50 (1.89)	−5.49 (1.45)	−.01 (−4.69, 4.67)	.997	.00 (−.45, .45)
**Secondary outcomes**										
**Vineland‐3 composite score**	2.90 (1.05)	.92 (.81)	1.98 (−.62, 4.58)	.136	.34 (−.11, .80)	7.30 (1.33)	2.37 (1.02)	**4.93 (1.64, 8.22)**	.003	**.68 (.21, 1.14)**
Communication domain	3.83 (1.44)	1.88 (1.10)	1.95 (−1.60, 5.50)	.281	.25 (−.20, .70)	5.47 (1.64)	4.20 (1.26)	1.27 (−2.78, 5.32)	.538	.14 (−.31, .59)
Daily living skills domain	1.17 (1.91)	1.43 (1.47)	−.26 (−4.99, 4.46)	.913	−.02 (−.48, .43)	6.53 (1.64)	1.53 (1.26)	**5.00 (.95, 9.06)**	.016	**.56 (.10, 1.02)**
Socialisation domain	5.17 (1.97)	.86 (1.51)	4.30 (−.57, 9.18)	.084	.40 (−.06, .85)	1.17 (2.11)	3.73 (1.62)	**6.44 (1.23, 11.65)**	.015	**.56 (.10, 1.02)**
**ABC score**	−12.07 (3.92)	−11.24 (3.01)	−.83 (−1.51, 8.85)	.866	−.04 (−.49, .41)	−16.1 (3.86)	−18.14 (2.96)	2.04 (−7.49, 11.56)	.675	.10 (−.35, .55)
Sensory domain	−2.27 (1.22)	−.33 (.93)	−1.93 (−4.94, 1.07)	.208	−.29 (−.74, .16)	−2.60 (1.04)	−2.69 (.80)	.09 (−2.49, 2.66)	.948	.02 (−.44, .47)
Relating domain	−2.47 (1.26)	−3.00 (.97)	.53 (−2.58, 3.64)	.737	.08 (−.37, .53)	−3.30 (1.51)	−4.29 (1.16)	.99 (−2.75, 4.73)	.602	.12 (−.33, .57)
Body domain	−2.83 (1.21)	−.61 (.93)	−2.23 (−5.21, .76)	.144	−.33 (−.79, .12)	−4.43 (1.07)	−2.75 (.82)	−1.69 (−4.32, .94)	.209	−.29 (−.74, .17)
Language domain	−3.10 (1.19)	−3.33 (.91)	.23 (−2.70, 3.17)	.876	.04 (−.42, .49)	−2.70 (1.39)	−4.69 (1.07)	1.99 (−1.45, 5.42)	.257	.26 (−.19, .71)
Social and self‐help domain	−2.67 (1.02)	−1.84 (.78)	−.82 (−3.34, 1.70)	.522	−.15 (−.60, .30)	−3.70 (.93)	−3.37 (.71)	−.33 (−2.62, 1.96)	.779	−.07 (−.52, .39)

*Note*: Data are presented as least‐square means with standard errors after adjustment for sex, age and baseline Autism Diagnostic Observation Schedule score.

Abbreviations: ABC, Autism Behaviour Checklist; CI, confidence interval; FMT, faecal microbiota transplantation; SRS, Social Responsiveness Scale.

Among the 103 children with ASD, 19 experienced 28 AEs without group differences, and none withdrew from the trial owing to AEs. The proportion of reported AEs was similar between the FMT and placebo groups, with 12 (23.1%) and seven (13.7%) patients experiencing one or more AEs, respectively. The frequency of AEs was 18 and 10, respectively, indicating that the FMT group experienced more AEs. All participants’ AEs resolved or improved, with no serious AEs occurring (grade 3). The most common AE was fever, with the FMT group experiencing it four times and the placebo group five times (Table 3). Additionally, the FMT group appeared to have a higher occurrence of neurological/psychiatric AEs (FMT eight times vs. placebo two times; Table 3).

**TABLE 3:** Overview of adverse events in the FMT and placebo groups.

Characteristics	FMT (*n* = 52)	Placebo (*n* = 51)
**Any adverse events, *n* (%)**	12	7
**Withdrawal or unmasking, *n* (%)**	0	0
**Total adverse events (frequency)**	18	10
**Adverse events possibly related to FMT or placebo (frequency)**	15	6
**Adverse events severity (frequency)**		
Level 1	3	1
Level 2	12	5
Level 3	3	4
Level 4	0	0
Level 5	0	0
**Outcome for adverse events (frequency)**		
Adverse event leading to death	0	0
Resolved or improved	18	10
Resolved with sequelae	0	0
Not resolved during follow‐up or unknown course	0	0
**Organ‐specific adverse events (frequency)**		
Gastrointestinal	3	2
Neurological/psychiatric	8	2
Dermatology	2	1
Urinary	1	0
Nonspecific (fever)	4	5
**Timing of adverse events (frequency)**		
During the administration	4	1
During follow‐up within 2 weeks	9	4
During follow‐up >2 weeks	5	5
**Identification of adverse events (frequency)**		
Rash maculo‐papular	1	1
Fever	4	5
Constipation	0	1
Irritability	2	0
Insomnia	2	1
Restlessness	1	0
Lymph node pain	1	0
Depression	2	0
Urinary frequency	1	0
Diarrhoea	2	0
Mania	1	1
Vomiting	1	1

Abbreviation: FMT, faecal microbiota transplantation.

Our primary findings indicated that FMT did not significantly improve clinical symptoms in children with ASD; however, our analysis of secondary outcomes revealed that oral FMT improved the socialisation domain of the Vineland‐3 scale score. In addition, our results indicated a high tolerability of FMT in children with ASD. We also observed a significant placebo effect. Consequently, it is essential to conduct efficacy evaluations in ASD research using randomised, double‐blind, placebo‐controlled trials Figure 1.

**FIGURE 1:**
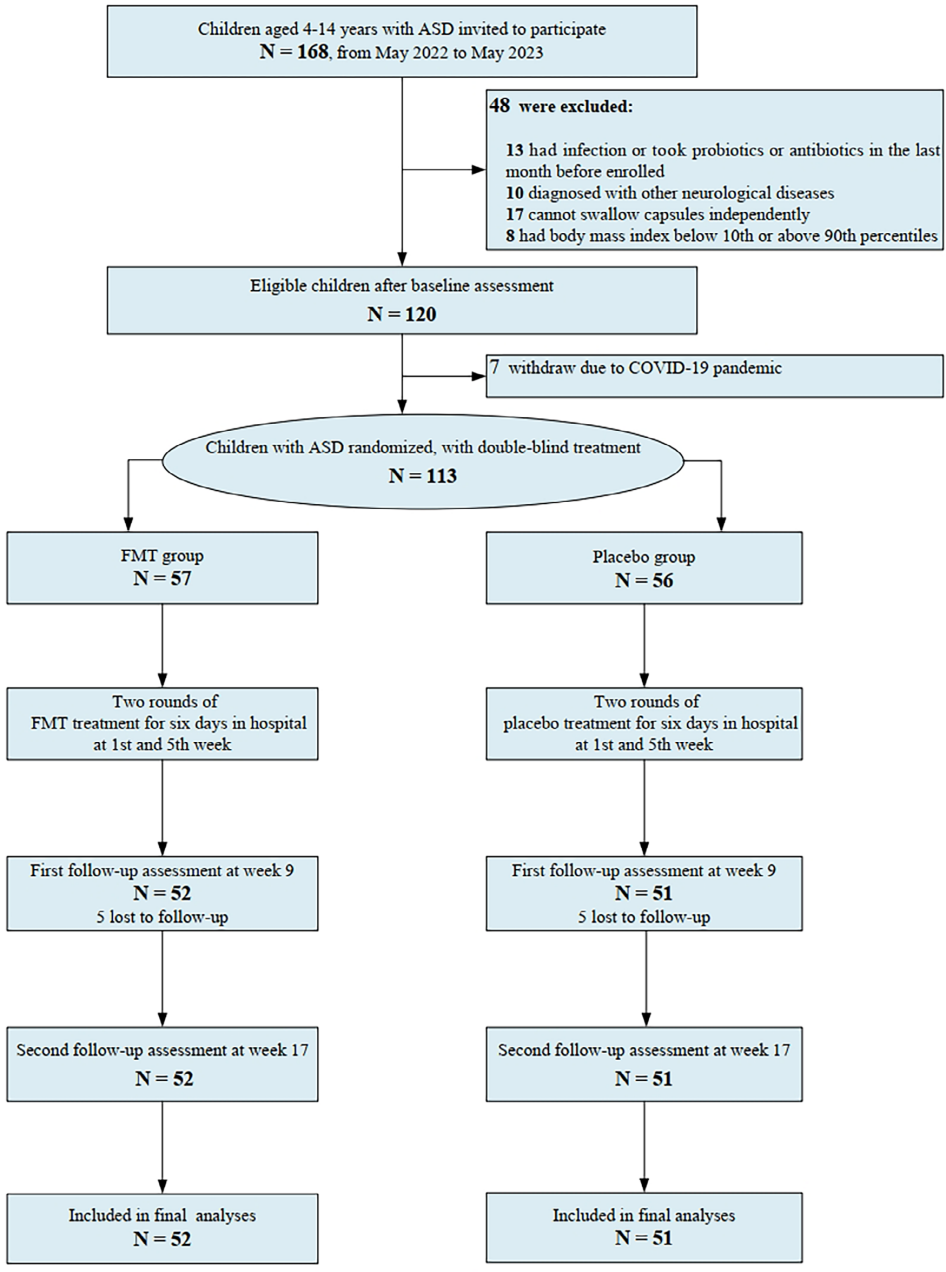
Participant flowchart.

## AUTHOR CONTRIBUTIONS

**Guang Yang; Zhiyong Zou** and **Xiaoyan Liu** were co‐responding authors and contributed equally to the conceptualisation; investigation and methodology of the study; and reviewed and edited the manuscript; had full access to all the data in the study and are responsible for the integrity of the data and the accuracy of the data analysis. **Xinyun Yao; Gang Zhu; Jun Cai; Guoyin Liu; Xinting Liu; Qianqian Niu; Siwen Li; Jing Gao; Jing Wang; Xiuyu Shi** and **Linyan Hu** contributed to the acquisition of data; investigation and methodology of the study and reviewed and edited the manuscript. **Lin Wan** contributed to investigation and methodology of the study; wrote the original draft; and reviewed and edited subsequent drafts of the manuscript. **Huan Wang** analysed the data; wrote the original draft; and reviewed and edited subsequent drafts of the manuscript. **Yan Liang** and **Xun Zhang** were involved in writing the original draft. **Bo Zhang** contributed to methodology of the study; and reviewed and edited the manuscript. All authors conceived the study; and revised and approved the final manuscript.

## CONFLICT OF INTEREST STATEMENT

No financial or non‐financial benefits were received or will be received from any party directly or indirectly related to the subject of this article.

## FUNDING INFORMATION

This research was funded by the general project of National Key Research and Development Program of China (References: 2023YFC2706405 and 2022YFC2705301), Beijing Natural Science Foundation (Reference: 7222187), the equipment comprehensive research project of General Armament Department, the key project of innovation cultivation fund of the Seventh Medical Center of Chinese PLA General Hospital (Reference: qzx−2023−1), the special scientific research project of Military Family Planning (Reference: 22JSZ20), and the National Natural Science Foundation of China (Reference: 82170302).

## ETHICS STATEMENT

We confirm that we have read the journal's position regarding issues involving ethical publication and affirm that this report is consistent with those guidelines.

## Supporting information

Supporting Information

## Data Availability

All data generated or analysed during this study are included in this published article and its Supporting Information files. Additional data that support the findings of this study are available via email from the corresponding author upon reasonable request.
