# Supplementary material for: Effect of oral faecal microbiota transplantation intervention for children with autism spectrum disorder: A randomised, double‐blind, placebo‐controlled trial
Source: Clin Transl Med. 2024 Aug 26;14(9):e70006. doi: 10.1002/ctm2.70006 (PMC11347384; doi:10.1002/ctm2.70006)
Supplement: Supplementary file 1 — Supporting Information [file CTM2-14-e70006-s001.docx]

**Supplementary Materials to: Effect of oral faecal microbiota transplantation intervention for children with autism spectrum disorder: a randomised, double-blind, placebo-controlled trial**

This Appendix provides further methodological details and additional Tables and Figures for “Effect of oral faecal microbiota transplantation intervention for children with autism spectrum disorder: a randomised, double-blind, placebo-controlled trial”

**Contents**

[Section 1: Appendix methods 2](#_Toc155072116)

[Methods 2](#_Toc155072117)

[The production process of the faecal microbiota transplantation (FMT) capsules 11](#_Toc155072117)

[Metagenomic sequencing 16](#_Toc155072118)

[Bioinformatics analysis 1](#_Toc155072119)7

[Section 2: Appendix Tables 19](#_Toc155072120)

[Table S1: Baseline characteristics of the participants 19](#_Toc155072121)

[Table S2: Baseline subdomain scores of the SRS-2, Vineland-3, and ABC of the participants 2](#_Toc155072121)1

[Table S3: Scores of the SRS-2, Vineland-3, and ABC by time points in the FMT and placebo groups 2](#_Toc155072122)4

[Table S4: Changes in the Vineland-3 subdomain scores in the FMT and placebo groups from baseline to week 9 and week 17 2](#_Toc155072123)6

[Table S5: Changes in the Vineland-3 subdomain scores in the FMT and placebo groups based on the colonisation rate at week 17 28](#_Toc155072124)

[Section 3: Appendix Figures 3](#_Toc155072125)0

[Figure S1: Changes in the Vineland-3 subdomain scores of the communication domain (A), daily living skills domain (B), and socialisation domain (C) in the FMT and placebo groups from baseline to week 9 and week 17 3](#_Toc155072126)1

[Figure S2: SRS-2 T-scores by time points in the FMT and placebo groups (a lower score indicates better outcomes) 3](#_Toc155072126)2

[Figure S3: Vineland-3 composite and domain scores by time points in the FMT intervention and placebo groups (a higher score indicates better outcomes) 3](#_Toc155072127)3

[Figure S4: ABC scores by time points in the FMT and placebo groups (a lower score indicates better outcomes) 3](#_Toc155072128)4

[Figure S5: Comparison of the gut microbiota alpha diversity (A) and beta diversity (B) pre- and post-treatment in the FMT and placebo groups 3](#_Toc155072128)5

[Section 4: References 3](#_Toc155072125)6

# Section 1: Appendix methods

## Methods

### Study design and participants

This randomised, double-blind, placebo-controlled clinical trial focussed on the oral administration of faecal microbiota capsules for the treatment of autism spectrum disorder (ASD). It was conducted at the Department of Paediatrics of the Chinese PLA General Hospital in Beijing, China. The trial followed the principles of good clinical practice and local regulations, with the protocol and any amendments approved by the Ethics Committee of Chinese PLA General Hospital (reference number S2021-631-01). Written informed consent was obtained from all participants’ caregivers. At the beginning of the trial, basic demographic information such as age, sex, family relationships, and self-rated anxiety scale (SAS) scores were collected. Additionally, chronic gastrointestinal symptoms (defined as symptoms lasting > 3 months according to the Gastrointestinal Symptom Rating Scale) and the Autism Diagnostic Observation Schedule, second edition (ADOS-2) scores of children with ASD were recorded. This study is registered at chictr.org.cn (ChiCTR2200057535).

Participants were recruited from May 2022 to May 2023. The inclusion criteria were as follows:
(1) Patients aged 4–14 years who were able to swallow capsules independently;
(2) Children diagnosed with ASD based on the Diagnostic and Statistical Manual of Mental Disorders, Fifth Edition (DSM-5) and International Classification of Diseases, 11th Revision (ICD-11) criteria;
(3) Biological parents who live with their children and can provide detailed assessment records.

The exclusion criteria were as follows:

(1) ASD with a known genetic condition (e.g., *MECP2-* or *MEF2C*-related neurodevelopmental disorders);

(2) Patients who used antibiotics, probiotics, or immunosuppressants within 1 month prior to the start of the trial;

(3) Patients who were following a special diet (e.g., ketogenic diet);

(4) Patients with body mass index (BMI) above the 90th sex- and age-specific percentiles or below the 10th percentile;

(5) Patients with the presence of respiratory or gastrointestinal infection symptoms within the past month (e.g., fever, cough, or diarrhoea);

(6) Patients with a history of inflammatory bowel diseases (e.g., ulcerative colitis or Crohn’s disease);

(7) Patients with a history of other significant neurological disorders (e.g., epilepsy, head trauma, encephalitis, or meningitis).

(8) Patients demonstrating a restricted eating pattern, characterised by a limited variety of food and only consuming one type of food. We administered a survey questionnaire that included nine food categories: staple food (rice/wheat), legumes, beans, vegetables, fruits, milk, meat, seafood, and eggs. Children who consumed < 6 of these food categories in the past month were excluded.

All participants had the freedom to withdraw from the trial at any time. The withdrawal criteria were as follows:

(1) Patients who failed to complete the entire faecal microbiota transplantation (FMT) treatment;
(2) Patients who failed to complete any of the follow-up assessments;
(3) Changes in previous psychiatric medications or adjustments of dosages during the trial period;
(4) Patients who required antibiotic treatment for infection symptoms during the trial.

Out of the initial 168 enrolled children, 113 were randomised, 10 discontinued their participation, and 103 completed the treatment. The trial started on May 1, 2022, and concluded on May 1, 2023. A total of 168 children with ASD were recruited for this study. Of these, 48 patients were excluded from the study: 13 had an infection or were taking antibiotics in the month before enrolment, 10 had other neurological diseases, 8 had a BMI below the 10th or above the 90th percentile, and 17 were unable to swallow capsules independently. Seven patients withdrew due to the coronavirus disease 2019 (COVID-19) pandemic. As a result, 113 patients were randomly assigned to either the FMT group or the placebo group, and ultimately, 103 patients (85.8%) completed two rounds of treatment and follow-up assessments at week 9 and week 17 (**Figure 1**).

### Randomisation and masking

### All study subjects were randomly assigned to receive either oral faecal microbiota capsules or placebo capsules at a 1:1 ratio. Randomisation was conducted using the randomizr package in the R language. Each patient was assigned a random number and the corresponding treatment package (containing either FMT or placebo capsules) without any visual distinction between the two interventions. Participants and researchers were blinded to the intervention assignment as personnel not involved in the study allocated random numbers and prepared the treatment packages. All participants were instructed to take capsules from their treatment package that matched their assigned random number. Throughout the study, researchers conducted efficacy and safety assessments, which were recorded in an electronic folder named after each participant’s assigned number. The group assignment information of each child was strictly kept confidential during the trial process. This confidentiality extended to the patients, caregivers, and researchers, who did not have access to the participant’s group assignment information until the completion of the study at week 17, when the data were locked. Subsequently, the group assignment information was disclosed.

### Procedures

All eligible participants who were enrolled and randomised to either FMT or placebo received the assigned agent during both 6-day administration periods in the hospital. Participants were instructed to follow a semi-liquid, low-residue diet for 3 days prior to the treatment. On the day of the trial, they were instructed to take the assigned capsules at 9:00 AM without eating breakfast. Before taking the capsules, participants were also instructed to consume intermittent oral electrolyte solution from 8:00 PM the night before the trial day until 5:00 AM on the trial day, until they expelled clear and transparent liquid. During the first 6-day treatment, participants took eight capsules with room temperature water before breakfast each day. After taking the capsules, participants received 1-hour observations and instructions regarding their daily diet. The second round of treatment was administered during the 5th week of the trial, and the procedures were the same as those in the first round, except for the gut preparation (the semi-liquid diet and oral electrolyte solutions). Patients were not hospitalised between treatments. Caregivers were asked not to change participants’ dietary habits throughout the entire experimental period.

The faecal microbiota capsules were produced according to the international guidelines.^1,2^ Approximately 100–200 mg of faeces was extracted from five healthy donors to yield approximately 50–80 capsules. Each FMT capsule consisted of 80 mg of freeze-dried faecal microbiota. Detailed information about the donor selection and the capsule manufacturing method is available in the section relating to the **production process of the faecal microbiota transplantation (FMT) capsules**. A single patient received faecal microbiota content from the same healthy donor. The placebo capsules, resembling the faecal microbiota capsules in appearance, smell, and weight, were filled with microcrystalline cellulose. Efficacy outcomes were assessed at baseline and prespecified visits, with the first post-dose assessment in the 9th week and the second assessment in the 17th week.

### Outcomes

The primary outcome measured in this study was the difference in the change in the SRS-2 T-score, which includes five domains: social awareness, social cognition, social communication, social motivation, and behavioural styles. This measurement was taken between groups from baseline to the end of week 9 and week 17. The SRS-2 T-scores ranged 32–114 points, with higher scores indicating more severe ASD symptoms.
The secondary outcomes included comparing the changes in scores on the Vineland-3 scale between the groups. This scale has three domains with corresponding subdomains: communication (receptive, expressive, and written), daily living skills (personal, domestic, and community), and socialisation (interpersonal relationships, play and leisure, and coping skills). The secondary outcomes also measured differences in scores on the ABC scale, which has five domains: sensory, relating, body, language, and social and self-help. These measurements were taken at baseline, the end of week 9, and the end of week 17. Higher Vineland-3 composite (standard score ranging 20–140) and subdomain scores (V-score ranging 1–24) indicate better adaptive behaviour, while higher ABC scale scores (ranging 0–149) reflect more severe autistic conditions.

All assessments were conducted by parents who were blinded to the treatment allocation. Researchers supervised and collected the evaluation questionnaires throughout the entire assessment process. Owing to the COVID-19 pandemic lockdown, follow-up assessments for some children were conducted through telephone interviews, although originally planned to be performed in outpatient settings.

Adverse events (AEs) and serious adverse events (SAEs) were defined according to the Common Terminology Criteria for Adverse Events (CTCAE) version 5.0 by the US Department of Health and Human Services. SAEs and suspected unexpected SAEs were reported to regulatory authorities. AEs were classified based on the CTCAE grading method from grade 1 (mild symptoms not requiring intervention) to grade 5 (death). They were further categorised as immediate complications during drug administration, events occurring within 24 h following drug administration, or events occurring > 24 hours after drug administration. Previous studies have noted that AEs related to FMT primarily affect the gastrointestinal system and can be classified into gastrointestinal and other system AEs.

### Process evaluation

All capsules were stored at -80°C in the hospital and removed at room temperature 3 h before oral administration. Children with ASD swallowed the assigned capsules independently in a separate consultation room or ward under the supervision of caregivers and researchers to ensure successful swallowing without chewing. Caregivers of participants were contacted through a smartphone application (WeChat) to inform them of the date of capsule ingestion, follow-up visits, and assessments. Caregivers also sent text messages or images to report any AEs to physicians and researchers in a timely manner. The strict supervision of capsule administration and timely feedback between caregivers and researchers ensured the successful execution of the trial. Furthermore, to evaluate changes in the gut microbiota following FMT, stool samples from all participants were collected before treatment and at the 5th week, 9th week, and 17th week after treatment. These samples were sequenced using the metagenomic shotgun method, and the colonisation rate among the FMT group at week 17 was computed to assess the success of FMT (see the section related to **metagenomic sequencing and bioinformatics analysis**).

### Sample size calculation

According to a previous study, a 10 mg dose of balovaptan led to a 3-point reduction in the total T-score on the SRS-2 scale compared with a placebo. We assumed that FMT would result in an average difference (δ) of 5 in the reduction in the total T-score of each domain of the SRS-2 compared with placebo at week 17. We used a pooled standard deviation (σ) of 3, a margin of superiority (MS) of 3, an α of 0.025, and a power of 0.90 to calculate the sample size, which resulted in 49 individuals per group, for a total of 98 individuals. Considering the relatively long follow-up period of this study, we accounted for a 15% dropout rate, resulting in a final sample size of 56 individuals per group, for a total of 112 individuals.

### Statistical analysis

We analysed the data using the per-protocol principle. We assessed differences in baseline characteristics between the FMT and placebo groups using the chi-square test for categorical variables and the Wilcoxon rank-sum or Student’s *t*-test for continuous variables. We also used the Student’s *t*-test to examine between-group differences in the SRS-2 T-score, Vineland-3 score, and ABC score at baseline, week 9, and week 17. We compared within-group paired changes between baseline and week 9 and week 17 using the paired *t*-test.

To assess the treatment effect, we used a mixed model for repeated measures to examine differences across the FMT and placebo groups in terms of mean changes in SRS-2, Vineland-3, and ABC scores from baseline to week 9 and from baseline to week 17. The repeated mixed-measure models included main effects for treatment (FMT vs. placebo), visit time (baseline, week 9, week 17), sex (boys vs. girls), age (continuous variable), and baseline ADOS-2 score (continuous variable), as well as the interaction between treatment and visit time. We retained all main effects and interactions in the final model regardless of their statistical significance. Visit time was fitted as a repeated effect with an unstructured correlation structure across visits within each participant. We calculated the least-square mean (LSM) with a 95% confidence interval (CI) and used the LSM with the corresponding standard error (SE) to convert the results into Cohen’s effect sizes for interpretation.

We conducted a sensitivity analysis based on the study by Chen et al., which suggests that the colonisation rate of incoming strains from the donor is associated with the efficacy of FMT in treating children with ASD.^5^ We compared the changes in the scores of the SRS-2 scale, Vineland-3 scale, and ABC scale between the FMT group with a donor gut microbiota colonisation rate of ≥ 20% and the placebo group, as well as between the FMT group with a donor gut microbiota colonisation rate of < 20% and the same placebo group.

We handled missing data using the multiple imputation method with the fully conditional specification method and chained equations. AEs were descriptively reported and summarised between the FMT and placebo groups. We used the Wilcoxon rank-sum test to compare the between-group differences in alpha diversity (Shannon, Simpson, Invsimpson, and Evenness) and beta diversity (Bray-Curtis distance) both pre- and post-treatment. We considered two-tailed p-values of < 0.05 to be statistically significant, and we adjusted the p-values for the microbiome analysis using the Benjamin-Hochberg false discovery rate method. All data analyses were performed using SAS 9.4 (SAS Institute, Cary, NC, USA) and R 4.2.2 (https://www.R-project.org/).

##

## The production process of the faecal microbiota transplantation (FMT) capsules

1. Stool donor screening

Healthy stool donors were screened based on international expert consensuses [6-10]. Since December 2019, because of the COVID-19 pandemic, all donors have been screened for COVID-19 infection by completing an epidemiological questionnaire and undergoing RT-PCR testing for SARS-CoV-2 using a nasopharyngeal swab before each donation. Stool testing for SARS-CoV-2 was not possible owing to the lack of a faecal SARS-CoV-2 testing platform.

**1.1 Questionnaire interview**

Initial Screening via Online Survey: A web-based survey was implemented to screen suitable faecal matter donors. The criteria for eligibility stipulate that donors must be robust individuals aged between 18–24 years with a BMI in the range of 18.5–23.9 kg/m^2^. Patients with histories of medical or lifestyle-related risk factors were excluded.

Criteria for inclusion:

- Completion of a comprehensive four-phase screening process, which included a survey, interview, physical checkup, and specific tests for urea, as well as blood and faecal analyses;
- Willingness and ability to provide informed consent for the donation of stool;
- Commitment to fill out and endorse the donation day questionnaire;
- Agreement to complete and sign a questionnaire designed to rule out COVID-19 infection.

Criteria for exclusion:

- Past medical conditions: Any prior infections (such HIV, hepatitis B/C, syphilis, HTLV-I/II, malaria, trypanosomiasis, and tuberculosis), gastrointestinal issues (such as irregular bowel movements and polyps), systemic immune or atopic conditions (including asthma, eczema, and eosinophilic gastrointestinal disorders), metabolic syndromes, significant undernourishment or obesity, chronic pain conditions (e.g., chronic fatigue syndrome and fibromyalgia), neuropsychiatric or neurodevelopmental disorders, any history of cancer, or psychological ailments.
- Medication usage: Consumption of antibiotics, antifungal or antiviral agents, probiotics, or laxatives in the past 3 months; use of immunosuppressants (such as calcineurin inhibitors, biologics, and corticosteroids) in the past 6 months; and intake of proton pump inhibitors for > 1 month.
- Family medical background: Three or more close relatives with gastrointestinal cancers or autoimmune/metabolic diseases; any relative currently having an active gastrointestinal infection.
- Vaccination records: Administration of any live attenuated virus vaccine or participation in experimental drug or vaccine trials within the last half-year.
- Recent travels: Visits to tropical regions or areas experiencing infectious disease outbreaks within the previous 6 months.
- Sexual practices: Engagement in high-risk sexual activities, including interactions with commercial sex workers, unknown individuals, or individuals with a history of drug use or sexually transmitted infections such as HIV, viral hepatitis, and syphilis.
- Lifestyle Choices: Use of tobacco, alcohol, or illicit substances.

**1.2 Mental Health Assessments**

- Patients with self-administered depression assessment scores > 52 were excluded.
- Those with scores > 49 on the SAS were excluded.
- Pittsburgh Sleep Quality Index scores > 10 warranted exclusion.

**1.3 Comprehensive Physical Assessment**

This assessment included an exhaustive physical examination of the heart, blood vessels, lungs, lymph nodes, eyes, ear-nose-throat, and oral cavity, all of which must show no abnormal findings.

**1.4 Diagnostic Laboratory Evaluations**

These tests were carried out within 3 weeks before stool donation and then periodically every 3–6 months [6, 11-15].

- Blood analysis (all results must be negative or within normal parameters): complete blood count, liver enzymes, bilirubin levels, gamma-glutamyl transferase, alkaline phosphatase, kidney function tests, including urea, creatinine, uric acid levels, fasting blood sugar, lipid profile, high-sensitivity C-reactive protein, electrolytes, and immunoglobulins. Viral screening for hepatitis A, B, C, D, E, HIV-1, HIV-2, Epstein-Barr virus, cytomegalovirus, rubella, herpes simplex virus, herpes simplex virus types 1 and 2, and syphilis. Additionally, tests for *Toxoplasma gondii* antibodies, *Caenorhabditis elegans*, and amoebae were performed.
- Stool analysis (all should be negative or within the normal range): Routine stool examination to check for consistency, colour, and the presence of undigested food, pus, mucus, parasites, and blood. Occult blood tests to detect blood hidden in the stool. Tests for pathogenic bacteria, including *Clostridium difficile, Campylobacter, Salmonella, Escherichia coli* O157:H7, *Shigella*, and Shiga toxin-producing *E. coli*. Screening for multiple drug-resistant bacteria, such as Vancomycin-resistant *Enterococcus*, carbapenem-resistant *Enterobacteriaceae*, β-lactamase-producing *E. coli*, and methicillin-resistant *Staphylococcus aureus*. Viral tests for norovirus type I, norovirus type II, rotavirus, and adenovirus antigens were also performed.
- Urine analysis: Urine tests for proteins, epithelial cells, white blood cells, red blood cells, pH, clarity (diaphaneity), colour, specific gravity, and glucose (urine sugar) were performed. All parameters needed to be within normal ranges, and the presence of any abnormalities could indicate underlying health issues that could exclude a potential donor.

**1.5. Other tests**

The C13 breath test must show no intestinal bacterial overgrowth and be negative for *H. pylori*. The SARS-CoV-2 nucleic acid nasal brush test should also be negative.

**2. FMT capsule manufacturing**

Donors provided stool specimens into a contaminant-free, sterile receptacle, ensuring no urine was present. The specimens were then promptly delivered to the lab at a temperature of 4–8ºC within 1 h. In the 10,000 cleanroom facilities of Oriental Yeekang (Beijing) Medicine Technology Co., Ltd., skilled staff created probiotic capsules under both anaerobic and aseptic conditions. Each donor sample was handled separately to prevent the combination of materials from various individuals.

- Each stool sample was assessed and weighed. Samples weighing < 100 g—indicative of potential gastrointestinal issues—or those showing signs of urine, mucus, or blood, or falling outside the Bristol Stool Chart’s type 3–5 range, were rejected.
- A roughly 1 mL portion of each specimen was sequestered into a sterile vial and immediately frozen at -80ºC for potential re-evaluation or tracking.
- The sample was blended with chilled sterile saline and gently homogenised for 2–3 min at low speed to preserve bacterial integrity.
- The mixture was sequentially strained through filters with diameters of 2.0, 1.0, 0.4, and 0.1 mm to eliminate bulk debris and fibrous material [15].
- Following 5-min centrifugation at 3000 g and 4ºC (using a Sorvall SS-34 rotor), the resulting pellet was resuspended in cryoprotective agents and then lyophilised according to previous protocols to produce a freeze-dried powder, which was then packed into acid-resistant size 3 hypromellose capsules (DRCaps from Capsugel) [16]. From each to 100–200 g stool sample from a single donor, about 50–100 size 3 FMT capsules are produced.
- Placebo capsules were constructed by filling identical size 3 DRCaps with comparable quantities of pharmaceutical microcrystalline cellulose(PH101, Linghu Xiwang Chemical Co., Huzhou, China).
- The finished capsules were stored at -80ºC.

##

## Metagenomic sequencing

**Sample preparation**

DNA was extracted from faecal samples using a Magnetic Soil and Stool DNA Kit (TIANGEN Biotech (Beijing) Co., Ltd., Beijing, China). Quality assessment and quantification of DNA were analysed using 1% agarose gel electrophoresis and Qubit® dsDNA Assay Kit in Qubit® 2.0 Fluorometer (Life Technologies, CA, USA). The sample DNA was diluted with sterile water until the OD value was between 1.8–2.0.

**Library construction, quality control, and sequencing**

First, 1 μg of genomic DNA was taken from the samples. Then, a NEBNext® Ultra DNA Library Prep Kit for Illumina (NEB, USA) was used to construct the library, while a Covaris ultrasonic fragmentation instrument was used to shear the DNA into 350 bp fragments. Library preparation was then performed after end-repair, A-tailing, and adapter ligation. Fragments containing adapters were PCR-amplified, size-selected, and purified. Next, the library was checked using Qubit and real-time PCR for quantification and a bioanalyzer for size distribution detection. The concentration was accurately quantified (the effective concentration of the library was > 3nM). The libraries were pooled and sequenced on an Illumina platform (PE150).

## Bioinformatics analysis

**Enhanced methodology for species annotation**

In the initial phase, sequencing produced ineffective sequences. These sequences often include adapter contaminants, repetitive segments, and low-quality reads, which can impede assembly and further analytical procedures. To mitigate this issue, we employed a rigorous filtering process for raw sequencing reads, which resulted in high-quality clean reads. Utilising these refined reads, we deployed Metaphlan2 software [17] for analytical purposes, leading to the generation of a comprehensive species abundance table across various taxonomic levels.

**Refined beta diversity analysis approach**

We employed the vegdist method from the vegan R package for beta diversity analysis, specifically using the ‘bray_curtis’ parameter. This method facilitates the calculation of the Bray-Curtis distance between pairs of samples. The formula used for this calculation was as follows:


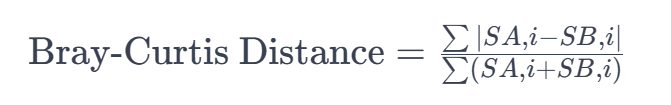


Where S,Ai, S,B,i, and i represent the relative abundance of the ith species in samples A and B, respectively.

**Colonisation rate analysis**

Our approach to calculating the colonisation rate was based on the methodology proposed by He et al. [18]. We first determined the distance of species i following FMT relative to the distance prior to FMT. Additionally, we calculated the distance of species i post-FMT compared with that of the donor. The formulas employed for these calculations were log(after/before + 1) and log(after/donor + 1). Here, ‘after’ denotes the relative abundance of species i post-transplantation, ‘before’ signifies its abundance pre-transplantation, and ‘donor’ refers to its abundance in the donor.

Species i was classified as a colonising bacterium when its post-transplantation distance from the recipient was less than its distance prior to transplantation (log(after/donor + 1) < log(after/before + 1)). Conversely, if the post-transplantation distance was greater, ((log(after/donor + 1) > log(after/before + 1)), species i was considered a native bacterium. The colonisation rate, denoted as C2R, was calculated using the following formula:


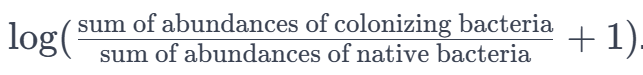


# Section 2: Appendix Tables

Table S1: Baseline characteristics of the participants

| **Characteristics** | **Total (N = 103)** | **FMT (n = 52)** | **Placebo (n = 51)** | **p-value** |
| --- | --- | --- | --- | --- |
| **Boys, n (%)** | 81 (78·6) | 42 (82·4) | 39 (75·0) | 0·363 |
| **Age, years** |  |  |  |  |
| Mean ± SD | 7·8 ± 2·0 | 7·7 ± 2·1 | 7·8 ± 2·0 | 0·819 |
| Median ± IQR | 7·0 ± 3·0 | 7·0 ± 3·0 | 7·0 ± 3·0 | 0·830 |
| Minimum to maximum | 5·0–13·0 | 5·0–13·0 | 5·0–13·0 | -- |
| **BMI, kg/m^2^** |  |  |  |  |
| Mean ± SD | 16·6 ± 3·4 | 16·6 ± 3·1 | 16·7 ± 3·7 | 0·787 |
| Median ± IQR | 15·7 ± 3·2 | 16·3 ± 3·3 | 15·4 ± 3·1 | 0·609 |
| Minimum to maximum | 11·2–28·3 | 11·2–28·0 | 12·5–28·3 | -- |
| **Rater, n (%)** |  |  |  | 0·592 |
| Father | 14 (13·6) | 6 (11·8) | 8 (15·4) |  |
| Mother | 89 (86·4) | 45 (88·2) | 44 (84·6) |  |
| **Self-rated anxiety scale of rater** |  |  |  |  |
| Mean ± SD | 33·7 ± 7·6 | 33·5 ± 7·2 | 33·9 ± 8·1 | 0·790 |
| Median ± IQR | 33·0 ± 11·0 | 33·0 ± 11·0 | 33·0 ± 11·0 | 0·987 |
| Minimum to maximum | 20·0–53·0 | 20·0–49·0 | 22·0–53 | -- |
| **Chronic gastrointestinal symptoms, n (%)** | 60 (58·3) | 29 (56·9) | 31 (59·6) | 0·777 |
| **ADOS** |  |  |  |  |
| Mean ± SD | 8·5 ± 1·7 | 8·5 ± 1·8 | 8·4 ± 1·7 | 0·747 |
| Median ± IQR | 9·0 ± 2·0 | 9·0 ± 2·0 | 9·0 ± 3·0 | 0·622 |
| Minimum to maximum | 2·7–10·0 | 2·7–10·0 | 4·0–10·0 | -- |
| **SRS-2 T-score** |  |  |  |  |
| Mean ± SD | 78·7 ± 9·7 | 78·3 ± 9·9 | 79·1 ± 9·6 | 0·688 |
| Median ± IQR | 79·7 ± 13·0 | 79·4 ± 10·0 | 80·0 ± 15·0 | 0·799 |
| Minimum to maximum | 48·0–99·0 | 48·0–98·0 | 55·0–99·0 | -- |
| **Vineland-3 composite score** |  |  |  |  |
| Mean ± SD | 66·8 ± 12·2 | 67·4 ± 13·0 | 66·2 ± 11·5 | 0·618 |
| Median ± IQR | 67·0 ± 14·0 | 67·0 ± 15·0 | 67·0 ± 13·0 | 0·872 |
| Minimum to maximum | 36·0–104·0 | 36·0–104·0 | 37·0–97·0 | -- |
| **ABC score** |  |  |  |  |
| Mean ± SD | 69·5 ± 25·4 | 68·9 ± 26·9 | 70·1 ± 23·9 | 0·816 |
| Median ± IQR | 68·0 ± 40·0 | 67·5 ± 35·5 | 72·0 ± 41·0 | 0·709 |
| Minimum to maximum | 17·0–133·0 | 17·0–133·0 | 23·0–123·0 | -- |

: Social Responsiveness Scale.

## Table S2: Baseline subdomain scores of the SRS-2, Vineland-3, and ABC of the participants

| **Scores** | **Total (N = 103)** | **FMT (n = 52)** | **Placebo (n = 51)** | **p-value** |
| --- | --- | --- | --- | --- |
| **Social Responsiveness Scale-2** |  |  |  |  |
| **Consciousness domain** |  |  |  |  |
| Mean ± SD | 69·2 ± 12·2 | 68·6 ± 12·4 | 69·8 ± 12·2 | 0·606 |
| Median ± IQR | 70·0 ± 16·0 | 71·0 ± 19·0 | 70·0 ± 17·0 | 0·784 |
| Minimum to maximum | 42·0–103·0 | 42·0–103·0 | 42·0–96·0 | -- |
| **Cognitive** **domain** |  |  |  |  |
| Mean ± SD | 76·6 ± 9·9 | 76·4 ± 10·5 | 76·7 ± 9·3 | 0·885 |
| Median ± IQR | 77·0 ± 13·0 | 76·1 ± 9·5 | 77·0 ± 13·0 | 0·895 |
| Minimum to maximum | 42·0–98·0 | 42·0–94·0 | 59·0–98·0 | -- |
| **Communication domain** |  |  |  |  |
| Mean ± SD | 78·6 ± 10·5 | 78·5 ± 10·9 | 78·8 ± 10·2 | 0·911 |
| Median ± IQR | 79·0 ± 12·0 | 80·0 ± 13·0 | 79·0 ± 12·0 | 0·968 |
| Minimum to maximum | 48·0–102·0 | 48·0–101·0 | 51·0–102·0 | -- |
| **Motivation domain** |  |  |  |  |
| Mean ± SD | 71·1 ± 10·8 | 71·1 ± 11·1 | 71·1 ± 10·5 | 0·999 |
| Median ± IQR | 71·0 ± 17·0 | 73·0 ± 19·0 | 71·0 ± 17·0 | 0·900 |
| Minimum to maximum | 38·0–91·0 | 38·0–91·0 | 51·0–89·0 | -- |
| **Behavioural styles domain** |  |  |  |  |
| Mean ± SD | 77·1 ± 10·1 | 76·3 ± 9·8 | 78·0 ± 10·4 | 0·406 |
| Median ± IQR | 77·0 ± 16·0 | 76·0 ± 16·5 | 80·0 ± 16·0 | 0·464 |
| Minimum to maximum | 55·0–105·0 | 55·0–96·0 | 57·0–105·0 | -- |
| **Vineland-3** |  |  |  |  |
| **Communication domain** |  |  |  |  |
| Mean ± SD | 65·3 ± 18·3 | 66·4 ± 19·8 | 64·1 ± 16·8 | 0·533 |
| Median ± IQR | 68·0 ± 22·0 | 70·5 ± 21·0 | 67·0 ± 26·0 | 0·458 |
| Minimum to maximum | 20·0–105·0 | 20·0–105·0 | 20·0–100·0 | -- |
| **Receptive subdomain** |  |  |  |  |
| Mean ± SD | 9·0 ± 3·8 | 9·1 ± 4·0 | 9·0 ± 3·6 | 0·918 |
| Median ± IQR | 10·0 ± 5·0 | 10·0 ± 5·0 | 9·0 ± 5·0 | 0·799 |
| Minimum to maximum | 1·0–16·0 | 1·0–16·0 | 1·0–15·0 | -- |
| **Expressive subdomain** |  |  |  |  |
| Mean ± SD | 8·5 ± 3·6 | 8·7 ± 4·1 | 8·3 ± 3·2 | 0·564 |
| Median ± IQR | 8·0 ± 5·0 | 9·0 ± 4·0 | 8·0 ± 5·0 | 0·512 |
| Minimum to maximum | 1·0–19·0 | 1·0–19·0 | 1·0–15·0 | -- |
| **Written subdomain** |  |  |  |  |
| Mean ± SD | 8·7 ± 3·8 | 9·2 ± 4·0 | 8·3 ± 3·6 | 0·214 |
| Median ± IQR | 9·0 ± 6·0 | 9·0 ± 5·5 | 9·0 ± 5·0 | 0·230 |
| Minimum to maximum | 1·0–18·0 | 1·0–18·0 | 1·0–15·0 | -- |
| **Daily living skills domain** |  |  |  |  |
| Mean ± SD | 74·4 ± 14·1 | 75·8 ± 15·7 | 73·0 ± 12·2 | 0·308 |
| Median ± IQR | 74·0 ± 18·0 | 76·0 ± 21·5 | 74·0 ± 16·0 | 0·314 |
| Minimum to maximum | 42·0–129·0 | 42·0–129·0 | 50·0–116·0 | -- |
| **Personal subdomain** |  |  |  |  |
| Mean ± SD | 10·1 ± 2·8 | 10·4 ± 3·3 | 9·9 ± 2·2 | 0·324 |
| Median ± IQR | 10·0 ± 3·0 | 10·0 ± 4·0 | 9·0 ± 2·0 | 0·476 |
| Minimum to maximum | 4·0–20·0 | 4·0–20·0 | 6·0–18·0 | -- |
| **Domestic subdomain** |  |  |  |  |
| Mean ± SD | 12·0 ± 3·2 | 12·3 ± 3·5 | 11·7 ± 2·9 | 0·381 |
| Median ± IQR | 12·0 ± 3·0 | 12·0 ± 4·5 | 12·0 ± 3·0 | 0·602 |
| Minimum to maximum | 3·0–20·0 | 5·0–20·0 | 3·0–19·0 | -- |
| **Community subdomain** |  |  |  |  |
| Mean ± SD | 8·8 ± 3·6 | 9·0 ± 3·9 | 8·6 ± 3·3 | 0·544 |
| Median ± IQR | 9·0 ± 3·0 | 8·5 ± 3·5 | 9·0 ± 4·0 | 0·743 |
| Minimum to maximum | 1·0–23·0 | 2·0–23·0 | 1·0–17·0 | -- |
| **Socialisation domain** |  |  |  |  |
| Mean ± SD | 58·0 ± 16·6 | 57·3 ± 16·4 | 58·7 ± 16·9 | 0·675 |
| Median ± IQR | 58·0 ± 24·0 | 56·0 ± 25·0 | 62·0 ± 24·0 | 0·392 |
| Minimum to maximum | 22·0–100·0 | 30·0–100·0 | 22·0–87·0 | -- |
| **Interpersonal relationships subdomain** |  |  |  |  |
| Mean ± SD | 6·9 ± 3·0 | 6·9 ± 3·1 | 6·9 ± 2·9 | 0·975 |
| Median ± IQR | 7·0 ± 3·0 | 7·0 ± 3·0 | 7·0 ± 4·0 | 0·550 |
| Minimum to maximum | 1·0–15·0 | 1·0–15·0 | 1·0–13·0 | -- |
| **Play and leisure subdomain** |  |  |  |  |
| Mean ± SD | 7·3 ± 3·9 | 7·1 ± 4·2 | 7·5 ± 3·7 | 0·580 |
| Median ± IQR | 8·0 ± 6·0 | 7·0 ± 6·5 | 8·0 ± 5·0 | 0·414 |
| Minimum to maximum | 1·0–19·0 | 1·0–19·0 | 1·0–14·0 | -- |
| **Coping skills subdomain** |  |  |  |  |
| Mean ± SD | 7·9 ± 3·0 | 7·8 ± 3·0 | 8·0 ± 3·0 | 0·726 |
| Median ± IQR | 7·0 ± 5·0 | 7·0 ± 4·0 | 7·0 ± 5·0 | 0·572 |
| Minimum to maximum | 2·0–15·0 | 2·0–15·0 | 2·0–13·0 | -- |
| **Autism Behaviour Checklist score** |  |  |  |  |
| **Sensory domain** |  |  |  |  |
| Mean ± SD | 12·4 ± 5·9 | 12·5 ± 6·3 | 12·3 ± 5·5 | 0·913 |
| Median ± IQR | 13·0 ± 9·0 | 13·0 ± 8·5 | 13·0 ± 9·0 | 0·995 |
| Minimum to maximum | 0·0–25·0 | 0·0–25·0 | 3·0–24·0 | -- |
| **Relating domain** |  |  |  |  |
| Mean ± SD | 17·7 ± 8·2 | 17·7 ± 9·1 | 17·7 ± 7·3 | 0·975 |
| Median ± IQR | 17·0 ± 12·0 | 16·0 ± 12·0 | 18·0 ± 11·0 | 0·593 |
| Minimum to maximum | 0·0–39·0 | 0·0–39·0 | 0·0–32·0 | -- |
| **Body domain** |  |  |  |  |
| Mean ± SD | 8·8 ± 7·3 | 8·7 ± 7·4 | 8·9 ± 7·2 | 0·896 |
| Median ± IQR | 6·0 ± 10·0 | 6·0 ± 10·5 | 7·0 ± 10·0 | 0·786 |
| Minimum to maximum | 0·0–27·0 | 0·0–27·0 | 0·0–25·0 | -- |
| **Language domain** |  |  |  |  |
| Mean ± SD | 17·4 ± 6·4 | 16·8 ± 6·9 | 18·0 ± 5·9 | 0·355 |
| Median ± IQR | 17·0 ± 8·0 | 17·0 ± 6·5 | 18·0 ± 8·0 | 0·412 |
| Minimum to maximum | 0·0–29·0 | 0·0–29·0 | 6·0–28·0 | -- |
| **Social and self-help domain** |  |  |  |  |
| Mean ± SD | 13·5 ± 5·3 | 13·7 ± 5·6 | 13·3 ± 5·1 | 0·749 |
| Median ± IQR | 14·0 ± 7·0 | 13·5 ± 8·0 | 14·0 ± 9·0 | 0·704 |
| Minimum to maximum | 0·0–24·0 | 0·0–24·0 | 4·0–23·0 | -- |

, Social Responsiveness Scale.

## Table S3: Scores of the SRS-2, Vineland-3, and ABC by time points in the FMT and placebo groups

| **Scores** | **FMT (n = 52)** | | | | |  | **Placebo (n = 51)** | | | | |
| --- | --- | --- | --- | --- | --- | --- | --- | --- | --- | --- | --- |
|  | **Baseline** | **Week 9** | **Week 17** | **p-value^*^** | **p-value^#^** |  | **Baseline** | **Week 9** | **Week 17** | **p-value^*^** | **p-value^#^** |
| **SRS-2** | 78·33 ± 9·95 | 76·90 ± 10·18 | 74·59 ± 8·62 | 0·226 | 0·003 |  | 79·11 ± 9·60 | 77·30 ± 9·17 | 76·85 ± 9·04 | 0·133 | 0·052 |
| Consciousness domain | 68·58 ± 12·38 | 66·52 ± 11·46 | 66·07 ± 10·63 | 0·220 | 0·176 |  | 69·83 ± 12·19 | 67·94 ± 9·53 | 68·72 ± 10·15 | 0·278 | 0·552 |
| Cognition domain | 76·45 ± 10·51 | 75·26 ± 8·98 | 74·85 ± 8·46 | 0·315 | 0·266 |  | 76·73 ± 9·27 | 76·63 ± 9·23 | 76·63 ± 8·31 | 0·930 | 0·919 |
| Communication domain | 78·53 ± 10·94 | 78·75 ± 11·92 | 75·62 ± 9·61 | 0·884 | 0·045 |  | 78·77 ± 10·16 | 76·67 ± 10·15 | 77·02 ± 9·95 | 0·110 | 0·143 |
| Motivation domain | 71·15 ± 11·12 | 70·29 ± 11·85 | 67·20 ± 10·61 | 0·524 | 0·009 |  | 71·15 ± 10·50 | 70·27 ± 9·76 | 69·43 ± 14·64 | 0·442 | 0·384 |
| Behavioural styles domain | 76·31 ± 9·77 | 72·55 ± 11·48 | 70·67 ± 11·39 | 0·024 | 0·001 |  | 77·97 ± 10·42 | 75·77 ± 12·55 | 73·34 ± 11·36 | 0·155 | 0·004 |
| **Vineland-3 Composite** | 67·44 ± 12·95 | 68·95 ± 11·61 | 71·89 ± 15·51 | 0·156 | 0·001 |  | 66·24 ± 11·50 | 67·53 ± 11·89 | 68·61 ± 10·73 | 0·110 | 0·013 |
| **Communication domain** | 66·40 ± 19·81 | 68·64 ± 19·31 | 69·51 ± 19·85 | 0·113 | 0·048 |  | 64·14 ± 16·84 | 66·42 ± 15·7 | 68·33 ± 15·52 | 0·033 | 0·001 |
| Receptive | 9·06 ± 4·00 | 9·47 ± 3·47 | 9·86 ± 3·71 | 0·229 | 0·083 |  | 8·98 ± 3·59 | 9·52 ± 3·19 | 9·98 ± 3·69 | 0·126 | 0·022 |
| Expressive | 8·71 ± 4·06 | 8·77 ± 4·10 | 8·92 ± 4·41 | 0·825 | 0·439 |  | 8·29 ± 3·20 | 8·50 ± 3·08 | 9·06 ± 2·98 | 0·394 | 0·002 |
| Written | 9·19 ± 3·99 | 9·77 ± 4·18 | 9·62 ± 4·12 | 0·137 | 0·290 |  | 8·25 ± 3·60 | 8·73 ± 3·61 | 8·98 ± 3·48 | 0·027 | 0·007 |
| **Daily living skills domain** | 75·83 ± 15·68 | 75·53 ± 14·83 | 78·48 ± 16·96 | 0·876 | 0·090 |  | 72·98 ± 12·24 | 74·85 ± 14·13 | 74·51 ± 12·54 | 0·102 | 0·199 |
| Personal | 10·40 ± 3·28 | 10·09 ± 3·25 | 10·95 ± 3·20 | 0·509 | 0·186 |  | 9·86 ± 2·15 | 10·44 ± 3·44 | 10·31 ± 2·65 | 0·095 | 0·137 |
| Domestic | 12·27 ± 3·53 | 12·00 ± 2·98 | 12·81 ± 3·00 | 0·550 | 0·120 |  | 11·71 ± 2·94 | 11·69 ± 2·63 | 11·59 ± 2·67 | 0·948 | 0·704 |
| Community | 9·02 ± 3·90 | 9·43 ± 3·37 | 9·51 ± 4·22 | 0·377 | 0·323 |  | 8·59 ± 3·25 | 9·09 ± 3·48 | 9·18 ± 3·47 | 0·013 | 0·039 |
| **Socialisation domain** | 57·33 ± 16·37 | 61·26 ± 14·85 | 66·10 ± 18·95 | 0·046 | < 0·001 |  | 58·71 ± 16·94 | 59·97 ± 18·12 | 62·43 ± 16·24 | 0·411 | 0·035 |
| Interpersonal relationships | 6·92 ± 3·07 | 7·41 ± 2·47 | 8·19 ± 3·03 | 0·287 | 0·003 |  | 6·94 ± 2·94 | 7·42 ± 3·03 | 7·73 ± 2·51 | 0·065 | 0·026 |
| Play and leisure | 7·12 ± 4·23 | 7·71 ± 3·78 | 9·02 ± 4·54 | 0·232 | < 0·001 |  | 7·55 ± 3·66 | 7·44 ± 3·89 | 8·10 ± 3·75 | 0·758 | 0·153 |
| Coping skills | 7·75 ± 3·05 | 8·71 ± 2·85 | 9·33 ± 3·53 | 0·018 | 0·001 |  | 7·96 ± 3·03 | 8·31 ± 3·24 | 8·57 ± 3·07 | 0·364 | 0·136 |
| **ABC score** | 68·90 ± 26·90 | 56·29 ± 25·38 | 53·42 ± 23·21 | < 0·001 | < 0·001 |  | 70·06 ± 23·93 | 58·82 ± 27·73 | 51·92 ± 27·11 | < 0·001 | < 0·001 |
| Sensory domain | 12·46 ± 6·29 | 9·75 ± 6·20 | 9·40 ± 6·04 | 0·005 | 0·002 |  | 12·33 ± 5·52 | 12·00 ± 7·39 | 9·65 ± 7·08 | 0·721 | 0·001 |
| Relating domain | 17·65 ± 9·08 | 14·23 ± 8·66 | 14·44 ± 8·02 | 0·007 | 0·017 |  | 17·71 ± 7·34 | 14·71 ± 9·03 | 13·41 ± 8·98 | 0·003 | 0·001 |
| Body domain | 8·67 ± 7·42 | 6·38 ± 7·17 | 6·23 ± 6·73 | 0·013 | 0·006 |  | 8·86 ± 7·22 | 8·25 ± 7·01 | 6·12 ± 6·18 | 0·492 | 0·001 |
| Language domain | 16·79 ± 6·89 | 13·15 ± 7·58 | 13·10 ± 7·54 | 0·001 | 0·006 |  | 17·96 ± 5·85 | 14·63 ± 7·70 | 13·27 ± 7·57 | 0·001 | < 0·001 |
| Social and self-help domain | 13·65 ± 5·60 | 11·38 ± 5·79 | 10·23 ± 5·59 | 0·005 | 0·002 |  | 13·31 ± 5·13 | 11·47 ± 5·64 | 9·94 ± 6·03 | 0·014 | < 0·001 |

Data are presented as means ± standard deviation. * p-values estimated using a paired *t*-test for within-group differences from baseline to week 9; ^#^ p-values estimated using a paired *t*-test for within-group differences from baseline to week 17. ABC, Autism Behaviour Checklist; FMT, faecal microbiota transplantation; SRS, Social Responsiveness Scale.

## Table S4: Changes in the Vineland-3 subdomain scores in the FMT and placebo groups from baseline to week 9 and week 17

| **Scores** | **Baseline to week 9** | | | | |  | **Baseline to week 17** | | | | |
| --- | --- | --- | --- | --- | --- | --- | --- | --- | --- | --- | --- |
|  | **FMT**  **(n = 52)** | **Placebo**  **(n = 51)** | **Difference**  **(95% CI)** | **p-value** | **Cohen’s d**  **(95% CI)** |  | **FMT**  **(n = 52)** | **Placebo**  **(n = 51)** | **Difference**  **(95% CI)** | **p-value** | **Cohen’s d**  **(95% CI)** |
| **Communication domain** |  |  |  |  |  |  |  |  |  |  |  |
| Receptive subdomain | 0·41 (0·34) | 0·54 (0·35) | -0·13  (-1·09, 0·83) | 0·791 | -0·05  (-0·44, 0·33) |  | 0·81 (0·44) | 1·00 (0·44) | -0·19  (-1·41, 1·03) | 0·756 | -0·60  (-0·45, 0·33) |
| Expressive subdomain | 0·06 (0·26) | 0·21 (0·26) | -0·14  (-0·87, 0·58) | 0·697 | -0·08  (-0·47, 0·31) |  | 0·21 (0·25) | 0·76 (0·26) | -0·55  (-1·26, 0·16) | 0·127 | -0·30  (-0·69, 0·08) |
| Written subdomain | 0·57 (0·31) | 0·48 (0·31) | 0·10  (-0·76, 0·95) | 0·822 | 0·04  (-0·35, 0·43) |  | 0·43 (0·34) | 0·73 (0·34) | -0·30  (-1·23, 0·64) | 0·536 | -0·12  (-0·51, 0·26) |
| **Daily living skills domain** |  |  |  |  |  |  |  |  |  |  |  |
| Personal subdomain | -0·31 (0·41) | 0·58 (0·41) | -0·89  (-2·02, 0·24) | 0·125 | -0·31  (-0·69, 0·08) |  | 0·55 (0·36) | 0·45 (0·36) | 0·10  (-0·90, 1·09) | 0·848 | 0·04  (-0·35, 0·43) |
| Domestic subdomain | -0·27 (0·38) | -0·02 (0·38) | -0·25  (-1·30, 0·80) | 0·643 | -0·09  (-0·48, 0·29) |  | 0·54 (0·32) | -0·12 (0·33) | 0·66  (-0·24, 1·56) | 0·153 | 0·29  (-0·10· 0·67) |
| Community subdomain | 0·41 (0·36) | 0·50 (0·36) | -0·09  (-1·08, 0·90) | 0·864 | -0·04  (-0·42, 0·35) |  | 0·49 (0·40) | 0·59 (0·40) | -0·10  (-1·21, 1·01) | 0·859 | -0·04  (-0·42, 0·35) |
| **Socialisation domain** |  |  |  |  |  |  |  |  |  |  |  |
| Interpersonal relationships subdomain | 0·49 (0·37) | 0·48 (0·37) | 0·01  (-1·02, 1·04) | 0·985 | 0·00  (-0·38, 0·39) |  | 1·27 (0·37) | 0·78 (0·37) | 0·49  (-0·55, 1·52) | 0·355 | 0·19  (-0·20, 0·57) |
| Play and leisure subdomain | 0·60 (0·43) | -0·11 (0·44) | 0·71  (-0·49, 1·91) | 0·248 | 0·23  (-0·16, 0·62) |  | **1·91 (0·42)** | **0·55 (0·42)** | **1·36**  **(0·19, 2·53)** | **0·023** | **0·46**  **(0·06, 0·85)** |
| Coping skills subdomain | 0·96 (0·38) | 0·34 (0·39) | 0·61  (-0·46, 1·68) | 0·262 | 0·27  (-0·16, 0·61) |  | 1·58 (0·41) | 0·61 (0·42) | 0·98  (-0·17, 2·12) | 0·095 | 0·33  (-0·06, 0·72) |

## Data are presented as least-square means with standard errors after adjustment for sex, age, and baseline Autism Diagnostic Observation Schedule score. CI = confidence interval; FMT = faecal microbiota transplantation.

## Table S5: Changes in the Vineland-3 subdomain scores in the FMT and placebo groups based on the colonisation rate at week 17

| **Scores** | **Baseline to week 9** | | | | |  | **Baseline to week 17** | | | | |
| --- | --- | --- | --- | --- | --- | --- | --- | --- | --- | --- | --- |
|  | **FMT** | **Placebo**  **(n = 51)** | **Difference**  **(95% CI)** | **p-value** | **Cohen’s d**  **(95% CI)** |  | **FMT** | **Placebo**  **(n = 51)** | **Difference**  **(95% CI)** | **p-value** | **Cohen’s d**  **(95% CI)** |
| **Colonisation rate < 20% (n = 22 for FMT)** | |  |  |  |  |  |  |  |  |  |  |
| **Communication domain** |  |  |  |  |  |  |  |  |  |  |  |
| Receptive subdomain | 0·45 (0·58) | 0·31 (0·38) | 0·14  (-1·22, 1·50) | 0·839 | 0·05  (-0·45, 0·55) |  | 0·09 (0·69) | 1·00 (0·45) | -0·91  (-2·52, 0·70) | 0·268 | -0·28  (-0·78, 0·22) |
| Expressive subdomain | 0·00 (0·32) | 0·14 (0·21) | -0·14  (-0·9, 0·62) | 0·723 | -0·09  (-0·59, 0·41) |  | -0·27 (0·38) | 0·76 (0·25) | **-1·04**  **(-1·93, -0·15)** | 0·023 | **-0·58**  **(-1·09, -0·07)** |
| Written subdomain | -0·59 (0·44) | 0·49 (0·29) | **-1·08**  **(-2·12, -0·04)** | 0·041 | **-0·52**  **(-1·03, -0·02)** |  | -0·77 (0·47) | 0·73 (0·31) | **-1·50**  **(-2·61, -0·39)** | 0·008 | **-0·68**  **(-1·19, -0·17)** |
| **Daily living skills domain** |  |  |  |  |  |  |  |  |  |  |  |
| Personal subdomain | -0·55 (0·52) | 0·37 (0·34) | -0·92  (-2·15, 0·31) | 0·143 | -0·38  (-0·88, 0·13) |  | -0·59 (0·50) | 0·45 (0·33) | -1·04  (-2·22, 0·13) | 0·082 | -0·44  (-0·95, 0·06) |
| Domestic subdomain | -0·64 (0·47) | 0·04 (0·31) | -0·68  (-1·77, 0·42) | 0·225 | -0·31  (-0·81, 0·19) |  | -0·18 (0·50) | -0·12 (0·33) | -0·06  (-1·23, 1·10) | 0·914 | -0·03  (-0·53, 0·47) |
| Community subdomain | 0·41 (0·52) | 0·45 (0·34) | -0·04  (-1·27, 1·19) | 0·947 | -0·02  (-0·52, 0·48) |  | -0·27 (0·64) | 0·59 (0·42) | -0·86  (-2·35, 0·63) | 0·258 | -0·29  (-0·79, 0·22) |
| **Socialisation domain** |  |  |  |  |  |  |  |  |  |  |  |
| Interpersonal relationships subdomain | -0·73 (0·54) | 0·35 (0·35) | -1·08  (-2·34, 0·18) | 0·094 | -0·43  (-0·94, 0·07) |  | 0·14 (0·55) | 0·78 (0·36) | -0·65  (-1·95, 0·65) | 0·328 | -0·25  (-0·75, 0·25) |
| Play and leisure subdomain | 0·77 (0·76) | -0·29 (0·50) | 1·07  (-0·72, 2·85) | 0·241 | 0·30  (-0·21, 0·80) |  | 1·68 (0·66) | 0·55 (0·43) | 1·13  (-0·42, 2·68) | 0·152 | 0·37  (-0·14, 0·87) |
| Coping skills subdomain | 0·91 (0·57) | 0·43 (0·37) | 0·48  (-0·85, 1·81) | 0·482 | 0·18  (-0·32, 0·68) |  | 1·86 (0·66) | 0·61 (0·44) | 1·26  (-0·30, 2·81) | 0·114 | 0·40  (-0·10, 0·90) |
| **Colonisation rate ≥ 20% (n = 30 for FMT)** | |  |  |  |  |  |  |  |  |  |  |
| **Communication domain** |  |  |  |  |  |  |  |  |  |  |  |
| Receptive subdomain | 0·57 (0·44) | 0·31 (0·34) | 0·25  (-0·83, 1·33) | 0·646 | 0·11  (-0·34, 0·56) |  | 1·27 (0·55) | 1·00 (0·42) | 0·27  (-1·08, 1·61) | 0·698 | 0·09  (-0·36, 0·54) |
| Expressive subdomain | 0·03 (0·34) | 0·14 (0·26) | -0·10  (-0·95, 0·75) | 0·811 | -0·06  (-0·51, 0·39) |  | 0·3 (0·37) | 0·76 (0·28) | -0·46  (-1·37, 0·44) | 0·315 | -0·23  (-0·68, 0·22) |
| Written subdomain | 1·23 (0·37) | 0·49 (0·28) | 0·74  (-0·16, 1·65) | 0·108 | 0·37  (-0·09, 0·82) |  | 1·23 (0·38) | 0·73 (0·29) | 0·51  (-0·44, 1·45) | 0·293 | 0·24  (-0·21, 0·69) |
| **Daily living skills domain** |  |  |  |  |  |  |  |  |  |  |  |
| Personal subdomain | -0·03 (0·49) | 0·37 (0·38) | -0·41  (-1·61, 0·80) | 0·510 | -0·15  (-0·6, 0·3) |  | 1·4 (0·44) | 0·45 (0·34) | 0·95  (-0·14, 2·03) | 0·087 | 0·39  (-0·06, 0·85) |
| Domestic subdomain | 0·20 (0·46) | 0·04 (0·36) | 0·16  (-0·99, 1·31) | 0·783 | 0·06  (-0·39, 0·51) |  | 1·23 (0·41) | -0·12 (0·31) | **1·35**  **(0·35, 2·35)** | 0·008 | **0·65**  **(0·19, 1·11)** |
| Community subdomain | 0·47 (0·36) | 0·45 (0·28) | 0·02  (-0·88, 0·91) | 0·973 | 0·01  (-0·44, 0·46) |  | 1·10 (0·40) | 0·59 (0·31) | 0·51  (-0·48, 1·50) | 0·310 | 0·23  (-0·22, 0·68) |
| **Socialisation domain** |  |  |  |  |  |  |  |  |  |  |  |
| Interpersonal relationships subdomain | 1·30 (0·39) | 0·35 (0·30) | 0·95  (-0·02, 1·92) | 0·056 | 0·44  (-0·01, 0·9) |  | 2·27 (0·45) | 0·78 (0·34) | **1·48**  **(0·38, 2·59)** | 0·009 | **0·61**  **(0·15, 1·07)** |
| Play and leisure subdomain | 0·30 (0·51) | -0·29 (0·39) | 0·59  (-0·67, 1·85) | 0·355 | 0·21  (-0·24, 0·66) |  | 1·77 (0·53) | 0·55 (0·41) | 1·22  (-0·09, 2·53) | 0·069 | 0·42  (-0·04, 0·87) |
| Coping skills subdomain | 1·20 (0·49) | 0·43 (0·37) | 0·77  (-0·44, 1·97) | 0·211 | 0·29  (-0·16, 0·74) |  | 1·53 (0·51) | 0·61 (0·39) | 0·93  (-0·33, 2·18) | 0·147 | 0·33  (-0·12, 0·78) |

Data are presented as least-square means with standard errors after adjustment for sex, age, and baseline Autism Diagnostic Observation Schedule score. CI = confidence interval; FMT = faecal microbiota transplantation.

**Figure legends**

**Figure S1: Changes in the Vineland-3 subdomain scores of the communication domain (A), daily living skills domain (B), and socialisation domain (C) in the FMT and placebo groups from baseline to week 9 and week 17.** *Significant between-group differences are indicated by red dashed boxes. FMT = faecal microbiota transplantation.

**Figure S2: SRS-2 T-scores by time points in the FMT and placebo groups (a lower score indicates better outcomes).** The lines represent the raw unadjusted values, and the box-and-whisker plots represent the median (the middle line), the 75th and 25th percentiles (the upper and lower ends of the box, respectively), and the maximum and minimum values (the whiskers). Student’s *t*-test is used to compare group differences in the mean values at baseline, week 9, and week 17. Two-tailed p-values of < 0·05 are considered statistically significant. FMT = faecal microbiota transplantation; SRS = Social Responsiveness Scale.

**Figure S3: Vineland-3 scores by time points in the FMT and placebo groups (a lower score indicates better outcomes).** The lines represent the raw unadjusted values, and the box-and-whisker plots represent the median (the middle line), the 75th and 25th percentiles (the upper and lower ends of the box, respectively), and the maximum and minimum values (the whiskers). Student’s *t*-test is used to compare group differences in the mean values at baseline, week 9, and week 17. Two-tailed p-values of < 0·05 are considered statistically significant. FMT = faecal microbiota transplantation.

**Figure S4: ABC scores by time points in the FMT and placebo groups (a lower score indicates better outcomes).** The lines represent the raw unadjusted values, and the box-and-whisker plots represent the median (the middle line), the 75th and 25th percentiles (the upper and lower ends of the box, respectively), and the maximum and minimum values (the whiskers). Student’s *t*-test is used to compare group differences in the mean values at baseline, week 9, and week 17. Two-tailed p-values of < 0·05 are considered statistically significant. ABC = Autism Behaviour Checklist; FMT = faecal microbiota transplantation.

**Figure S5: Comparison of the gut microbiota alpha diversity (A) and beta diversity (B) pre- and post-treatment in the FMT and placebo groups.** The Wilcoxon rank-sum test is used to compare group differences. Two-tailed p-values of < 0.05 are considered statistically significant. FMT = faecal microbiota transplantation.

# Section 3: Appendix Figures


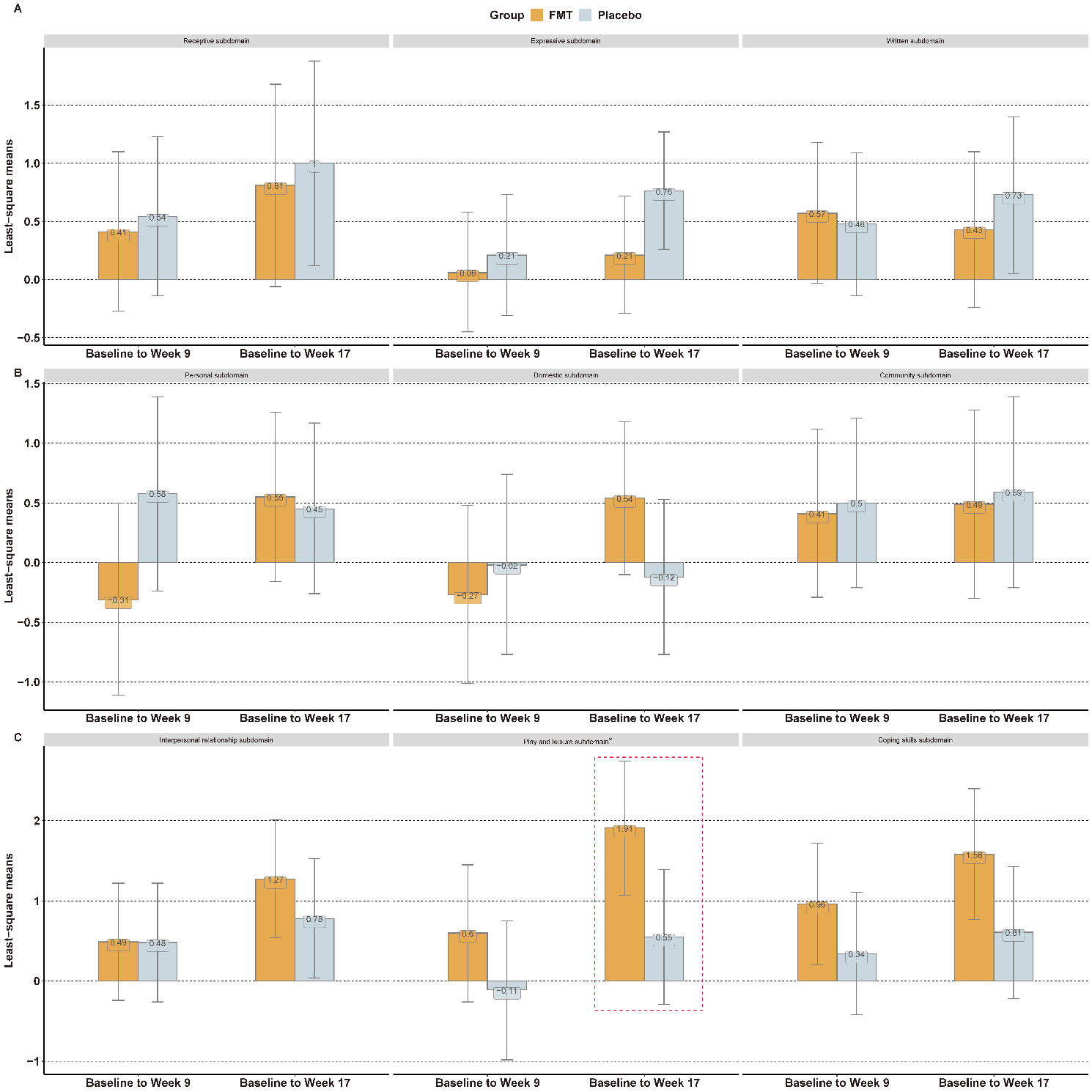


**Figure S1: Changes in the Vineland-3 subdomain scores of the communication domain (A), daily living skills domain (B), and socialisation domain (C) in the FMT and placebo groups from baseline to week 9 and week 17.** * Significant between-group differences are indicated by red dashed boxes. FMT = faecal microbiota transplantation.


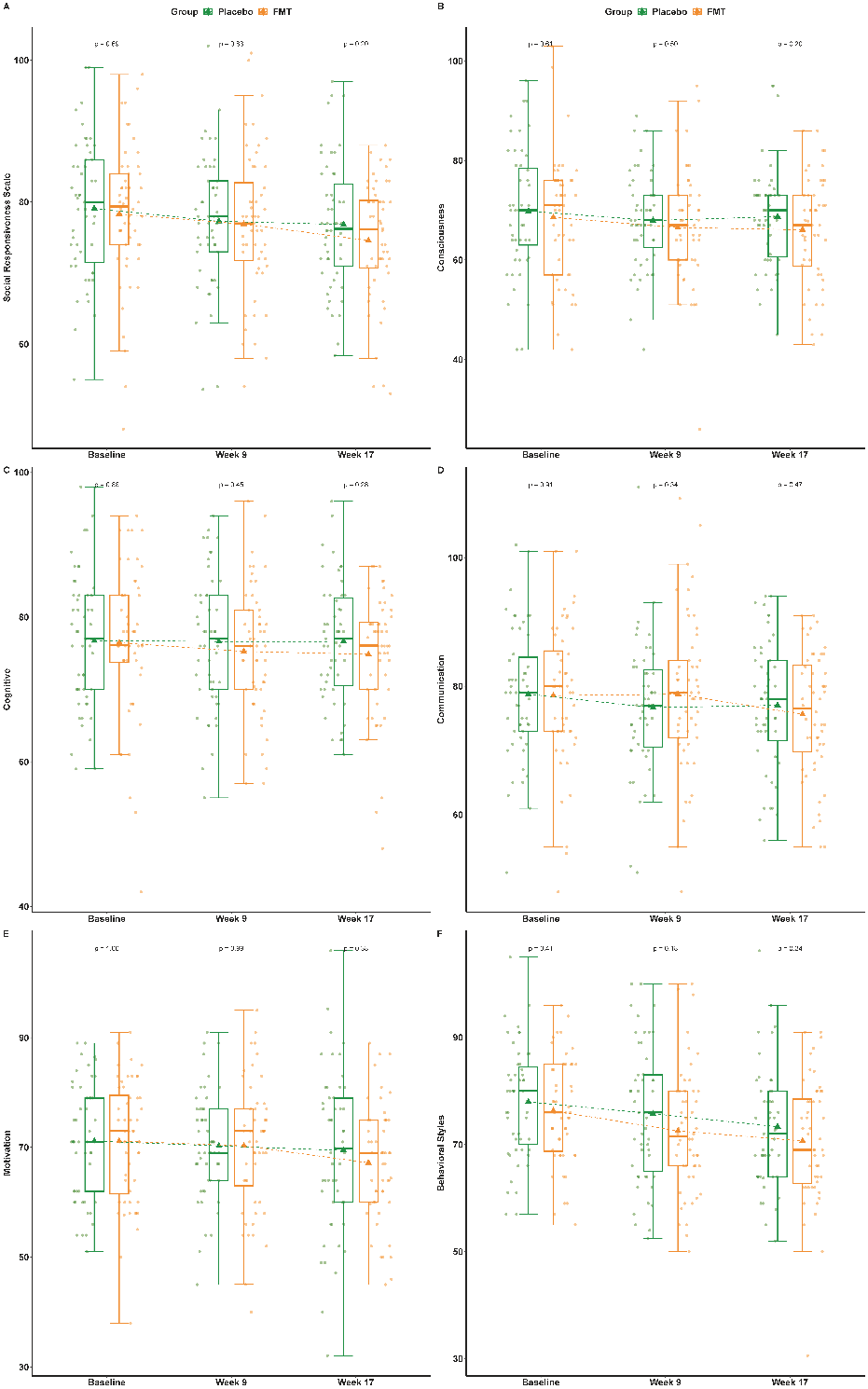


**Figure S2: SRS-2 T-scores by time points in the FMT and placebo groups (a lower score indicates better outcomes).** The lines represent the raw unadjusted values, and the box-and-whisker plots represent the median (the middle line), the 75th and 25th percentiles (the upper and lower ends of the box, respectively), and the maximum and minimum values (the whiskers). Student’s *t*-test is used to compare group differences in the mean values at baseline, week 9, and week 17. Two-tailed p-values of < 0·05 are considered statistically significant. FMT = faecal microbiota transplantation; SRS = Social Responsiveness Scale.

##
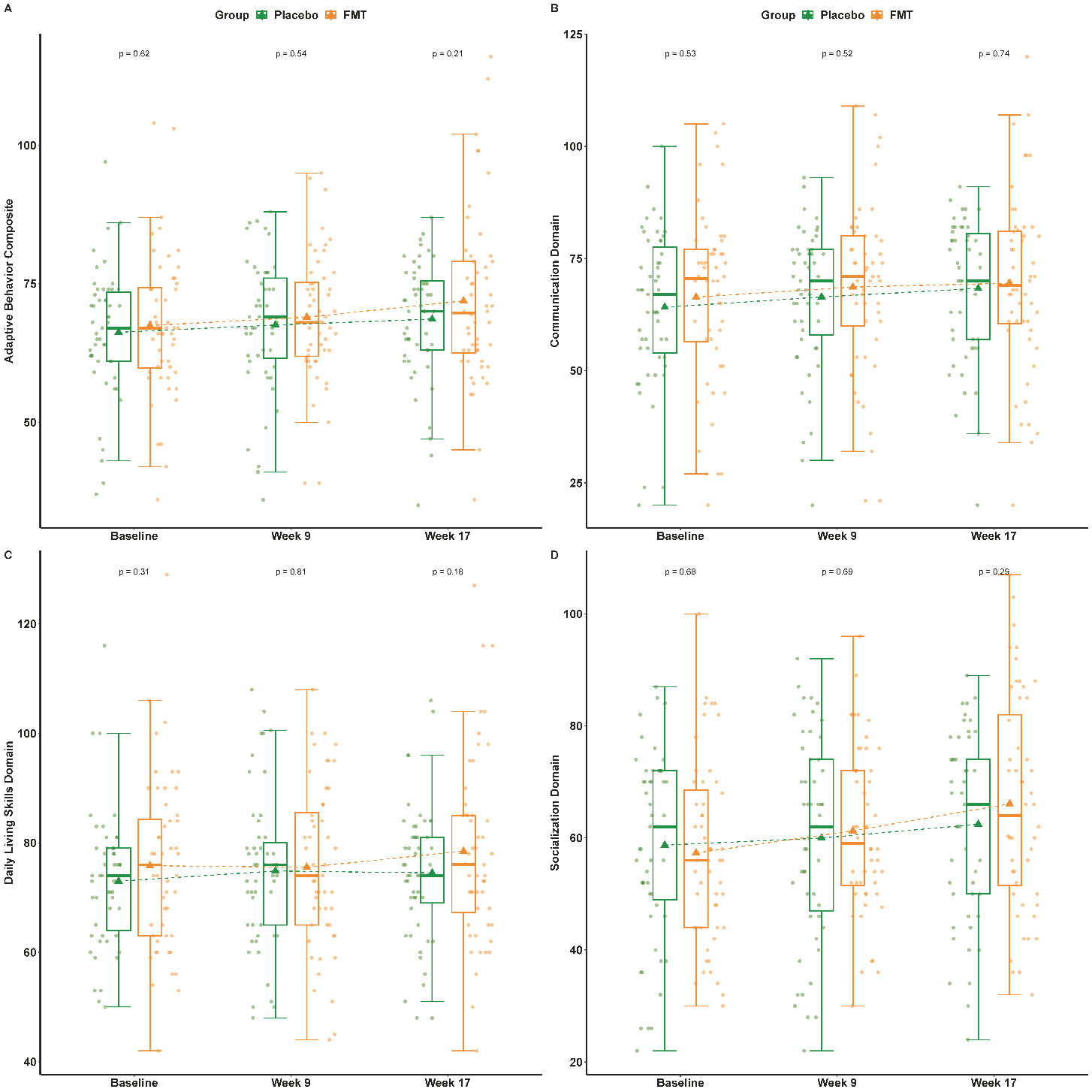


Figure S3: Vineland-3 composite and domain scores by time points in the FMT intervention and placebo groups (a higher score indicates better **outcomes).** The lines represent the raw unadjusted values, and the box-and-whisker plots represent the median (the middle line), the 75^th^ and 25^th^ percentiles (the upper and lower ends of the box, respectively), and the maximum and minimum values (the whiskers). Student’s *t*-test is used to compare group differences in the mean values at baseline, week 9, and week 17. Two-tailed p-values of < 0·05 are considered statistically significant. FMT = faecal microbiota transplantation.


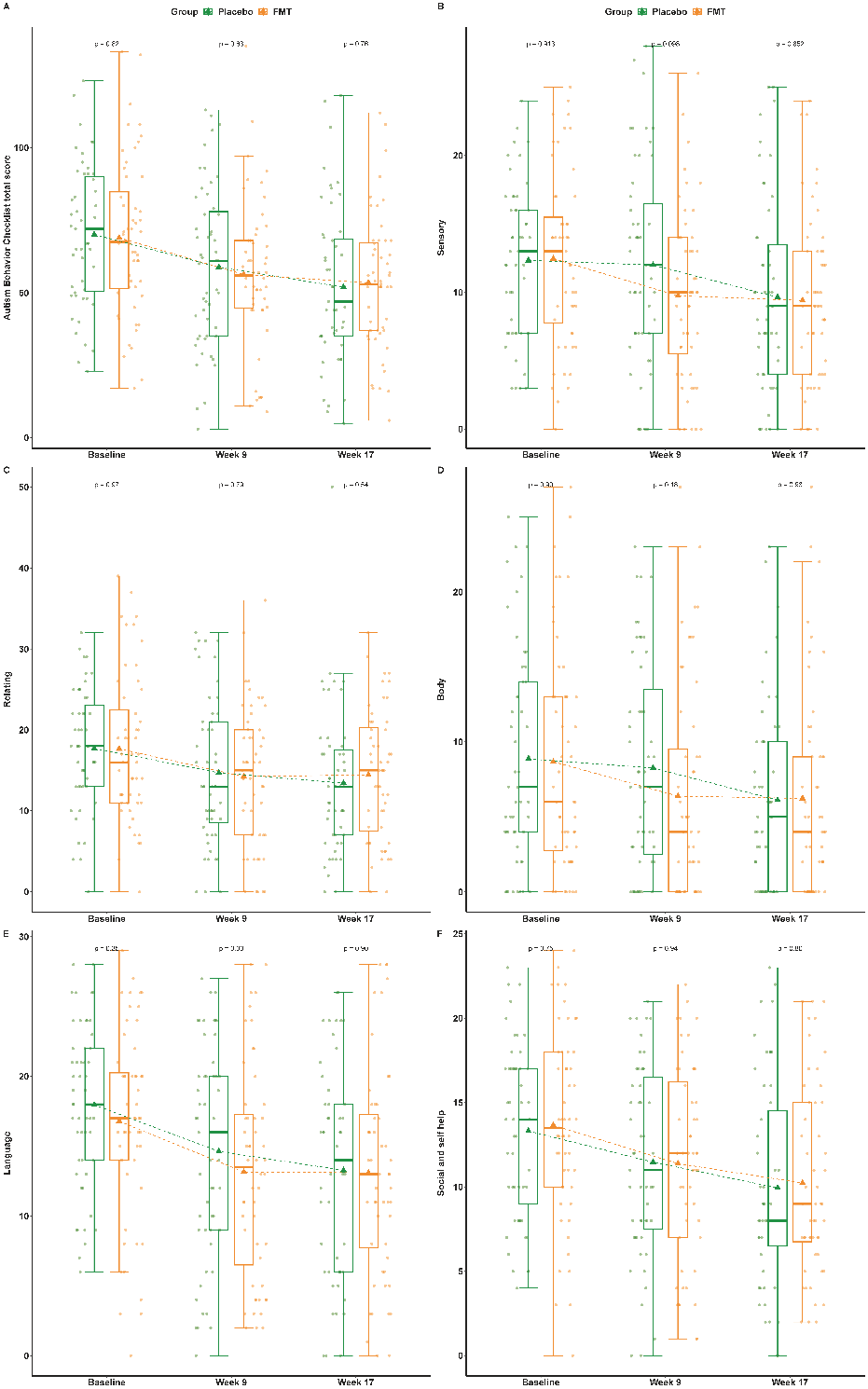


Figure S4: ABC scores by time points in the FMT and placebo groups (a lower score indicates better **outcomes).** The lines represent the raw unadjusted values, and the box-and-whisker plots represent the median (the middle line), the 75th and 25th percentiles (the upper and lower ends of the box, respectively), and the maximum and minimum values (the whiskers). Student *t*-test is used to compare group differences in the mean values at baseline, week 9, and week 17. Two-tailed p-values of < 0·05 are considered statistically significant. ABC = Autism Behaviour Checklist; FMT = faecal microbiota transplantation.


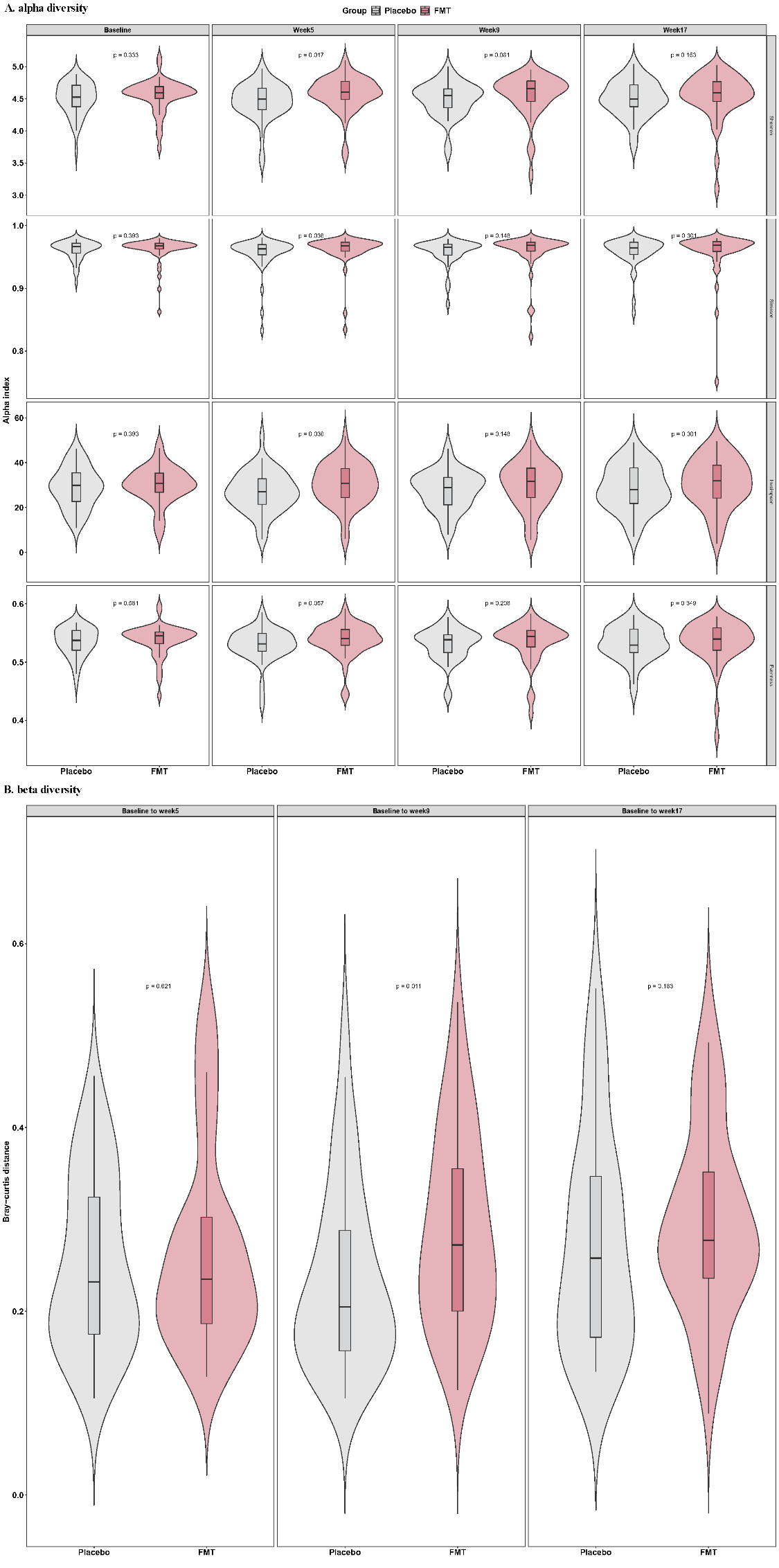


**Figure S5: Comparison of the gut microbiota alpha diversity (A) and beta diversity (B) pre- and post-treatment in the FMT and placebo groups.** The Wilcoxon rank-sum test is used to compare group differences. Two-tailed p-values of < 0.05 are considered statistically significant. FMT = faecal microbiota transplantation.

**References**

1. Cammarota G, Ianiro G, Kelly CR, et al. International consensus conference on stool banking for faecal microbiota transplantation in clinical practice. Gut. 2019;68(12):2111-2121.

2. Keller JJ, Ooijevaar RE, Hvas CL, et al. A standardised model for stool banking for faecal microbiota transplantation: a consensus report from a multidisciplinary UEG working group. United European Gastroenterol J. 2021;9(2):229-247.

3. Bolognani F, Del Valle Rubido M, Squassante L, et al. A phase 2 clinical trial of a vasopressin V1a receptor antagonist shows improved adaptive behaviors in men with autism spectrum disorder. Sci Transl Med. 2019;11(491):eaat7838 [pii].

4. Nakagawa S, Cuthill IC. Effect size, confidence interval and statistical significance: a practical guide for biologists. Biol Rev Camb Philos Soc. 2007;82(4):591-605.

5. Chen Q, Wu C, Xu J, et al. Donor-recipient intermicrobial interactions impact transfer of subspecies and fecal microbiota transplantation outcome. Cell Host Microbe. 2024;32(3):349-365.e4.

6. Nanjing consensus on methodology of washed microbiota transplantation, Chin Med J (Engl). 2020 Oct 5;133(19):2330-2332;

7. Consensus of Chinese experts on screening and management of fecal microbiota transplantation donors by the Subspecialty Committee of Parenteral and Enteral Nutrition, Chinese Medical Association and Microecology Professional Committee, Shanghai Preventive Medicine Association (2022 version) [J]. Chinese Journal of Gastrointestinal Surgery, 2022, 25(9):757-765. doi:10.3760/cmaj.cn441530-20220606-00246.

8. Cammarota, G., et al., International consensus conference on stool banking for faecal microbiota transplantation in clinical practice. Gut, 2019. 68(12): p. 2111-2121.

9. Haifer, C., et al., Australian consensus statements for the regulation, production and use of faecal microbiota transplantation in clinical practice. Gut, 2020. 69(5): p. 801-810.

10. Cammarota, G., et al., European consensus conference on faecal microbiota transplantation in clinical practice. Gut, 2017. 66(4): p. 569-580.

11. Rossen NG, Fuentes S, van der Spek MJ, Tijssen JG, Hartman JH, Duflou A, et al. Findings from a Randomized Controlled Trial of Fecal Transplantation for Patients with Ulcerative Colitis. Gastroenterology 2015; 149: 110-118 e114. doi: 10.1053/j.gastro.2015.03.045.

12. Tariq R, Weatherly R, Kammer P, Pardi DS, Khanna S. Donor Screening Experience for Fecal Microbiota Transplantation in Patients with Recurrent C. difficile Infection. J Clin Gastroenterol 2018; 52: 146-150. doi: 10.1097/MCG.0000000000000768.

13. Van Nood E, Vrieze A, Nieuwdorp M, Fuentes S, Zoetendal EG, de Vos WM, et al. Duodenal infusion of donor feces for recurrent Clostridium difficile. N Engl J Med 2013; 368: 407-415. doi: 10.1056/NEJMoa1205037.

14. Kump P, Wurm P, Grochenig HP, Wenzl H, Petritsch W, Halwachs B, et al. The taxonomic composition of the donor intestinal microbiota is a major factor influencing the efficacy of faecal microbiota transplantation in therapy refractory ulcerative colitis. Aliment Pharmacol Ther 2018; 47: 67-77. doi: 10.1111/apt.14387.

15. Xu F, Li N, Wang C, Xing H, Chen D and Wei Y. Clinical efficacy of fecal microbiota transplantation for patients with small intestinal bacterial overgrowth: a randomized, placebo-controlled clinic study. BMC GASTROENTEROL. 2021; 21(1):54.

16. Staley C, Hamilton MJ, Vaughn BP, Graiziger CT, Newman KM, Kabage AJ, Sadowsky MJ and Khoruts A. Successful Resolution of Recurrent Clostridium difficile Infection using Freeze-Dried, Encapsulated Fecal Microbiota; Pragmatic Cohort Study. AM J GASTROENTEROL. 2017; 112(6):940-947.

17. Truong DT, Franzosa EA, Tickle TL, Scholz M, Weingart G, Pasolli E, Tett A, Huttenhower C, Segata N. MetaPhlAn2 for enhanced metagenomic taxonomic profiling. Nat Methods. 2015 Oct;12(10):902-3. doi: 10.1038/nmeth.3589. Erratum in: Nat Methods. 2016 Jan;13(1):101. PMID: 26418763.

18. He R, Li P, Wang J, Cui B, Zhang F, Zhao F. The interplay of gut microbiota between donors and recipients determines the efficacy of fecal microbiota transplantation. Gut Microbes. 2022 Jan-Dec;14(1):2100197. doi: 10.1080/19490976.2022.2100197. PMID: 35854629; PMCID: PMC9302524.
